# The mRNA-bound proteome of the human malaria parasite *Plasmodium falciparum*

**DOI:** 10.1186/s13059-016-1014-0

**Published:** 2016-07-05

**Authors:** Evelien M. Bunnik, Gayani Batugedara, Anita Saraf, Jacques Prudhomme, Laurence Florens, Karine G. Le Roch

**Affiliations:** Department of Cell Biology and Neuroscience, University of California, Riverside, 900 University Avenue, Riverside, CA 92521 USA; Stowers Institute for Medical Research, 1000 E. 50th Street, Kansas City, MO 64110 USA

**Keywords:** Gene expression, Post-transcriptional regulation, Protein domains, RNA-binding proteins, Translation

## Abstract

**Background:**

Gene expression is controlled at multiple levels, including transcription, stability, translation, and degradation. Over the years, it has become apparent that *Plasmodium falciparum* exerts limited transcriptional control of gene expression, while at least part of *Plasmodium*’s genome is controlled by post-transcriptional mechanisms. To generate insights into the mechanisms that regulate gene expression at the post-transcriptional level, we undertook complementary computational, comparative genomics, and experimental approaches to identify and characterize mRNA-binding proteins (mRBPs) in *P. falciparum*.

**Results:**

Close to 1000 RNA-binding proteins are identified by hidden Markov model searches, of which mRBPs encompass a relatively large proportion of the parasite proteome as compared to other eukaryotes. Several abundant mRNA-binding domains are enriched in apicomplexan parasites, while strong depletion of mRNA-binding domains involved in RNA degradation is observed. Next, we experimentally capture 199 proteins that interact with mRNA during the blood stages, 64 of which with high confidence. These captured mRBPs show a significant overlap with the in silico identified candidate RBPs (*p* < 0.0001). Among the experimentally validated mRBPs are many known translational regulators active in other stages of the parasite’s life cycle, such as DOZI, CITH, PfCELF2, Musashi, and PfAlba1–4. Finally, we also detect several proteins with an RNA-binding domain abundant in Apicomplexans (RAP domain) that is almost exclusively found in apicomplexan parasites.

**Conclusions:**

Collectively, our results provide the most complete comparative genomics and experimental analysis of mRBPs in *P. falciparum.* A better understanding of these regulatory proteins will not only give insight into the intricate parasite life cycle but may also provide targets for novel therapeutic strategies.

**Electronic supplementary material:**

The online version of this article (doi:10.1186/s13059-016-1014-0) contains supplementary material, which is available to authorized users.

## Background

Malaria continues to contribute significantly to the global burden of disease, with an estimated 438,000 deaths and 214 million infected individuals in 2014 [1], the majority of which were caused by the most deadly human malaria parasite, *Plasmodium falciparum*. Despite continued efforts aimed at preventing infections, treatment of infected individuals is still an essential part of the strategy to reduce malaria morbidity and mortality. Given the importance of treatment in the control of malaria, the spread of drug-resistant parasites is alarming [2, 3] and calls for the development of novel antimalarial drugs.

Since the completion of the *P. falciparum* genome over a decade ago [4], much effort has been put into deciphering patterns of gene expression in the parasite, motivated by the notion that this will increase our understanding of parasite biology and reveal attractive targets for novel antimalarial drugs [5–7]. In addition, the process of gene regulation is governed by essential regulatory components that by themselves could be novel drug targets.

Gene expression is controlled at multiple levels by means of mechanisms that regulate gene transcription or that act post-transcriptionally to affect the stability or translational efficiency of the transcript. Over the years, it has become apparent that *P. falciparum* exerts limited control of gene expression at the level of transcription. The number of transcription-associated proteins, such as specific transcription factors and subunits of the mediator complex, is relatively low in both *P. falciparum* and the second most prevalent human malaria parasite, *P. vivax*, as compared to other eukaryotes [8–11]. In addition, strong epigenetic control of gene expression is only observed for several gene families involved in antigenic variation [12, 13]. On the other hand, various studies have found discrepancies between steady-state mRNA levels and protein abundance or levels of protein synthesis, with a delay in translation for a subset of genes [14–17], suggesting that at least part of *Plasmodium*’s genome is controlled by post-transcriptional mechanisms.

Post-transcriptional mechanisms of gene regulation are centered around RNA-binding proteins (RBPs), several of which have been shown to play important roles in parasite biology, in particular during the transmission stages. *Plasmodium* species lack homologs of the RNA interference machinery [18], and mechanisms of post-transcriptional control that have thus far been identified in the parasite are based on translational repression by stabilization and storage of transcripts. In sporozoites, an RBP of the Pumilio/FBF family PUF2 (PF3D7_0417100) is essential for maintaining translational repression resulting in latency [19–22]. In female gametocytes, hundreds of transcripts are translationally repressed during the transformation into ookinetes in the mosquito midgut [23]. The ATP-dependent RNA helicase DDX6 (DOZI; PF3D7_0320800) and a homolog of CAR-I in fly and Trailer Hitch in worm (CITH; PF3D7_1474900) regulate the storage of these transcripts into ribonucleoprotein complexes in the cytoplasm of the female gametocyte [24, 25]. In addition, PUF2 represses the translation of a number of gametocyte transcripts [26], but it does not seem to be present in the DOZI- and CITH-dependent RNA granules.

Several RBPs have been shown to be involved in post-transcriptional regulation of gene expression during the intraerythrocytic developmental cycle (IDC). PfCAF1 (PF3D7_0811300) and PfAlba1 (PF3D7_0814200) both regulate hundreds of transcripts and are particularly important for stabilization of transcripts encoding egress and invasion proteins [27, 28]. In addition, PfSR1 (PF3D7_0517300) controls alternative splicing and transcript abundance for a subset of genes [29]. However, little is known about other RBPs that are expressed during the IDC and their role in mRNA homeostasis.

A recent bioinformatics analysis by Reddy et al*.* cataloged RBPs with the common RNA recognition motif (RRM) and RNA helicase motifs, as well as several other less common RNA-binding domains (RBDs) [30]. However, many additional RNA-binding motifs have been identified in other eukaryotic genomes. We therefore undertook a comprehensive computational and comparative genomics approach to generate an extended atlas of RBPs in *P. falciparum*. In addition, we provide experimental evidence for a role of a subset of these RBPs during the IDC of the parasite. Our results validate that mechanisms regulating translation are most likely complex. A better understanding of these regulatory RBPs will not only provide insights into the intricate life cycle of this deadly parasite, but will also assist the identification of novel targets for therapeutic strategies.

## Results

### Identification and classification of RNA-binding proteins in *P. falciparum*

To characterize the repertoire of RNA-binding proteins (RBPs) in *P. falciparum*, we performed a hidden Markov model (HMM) search on the parasite proteome using 793 domains from the protein family (Pfam) database that are known to interact with RNA or that are found in RNA-related proteins (Additional file 1). These domains cover the complete range of RNA-related cellular functions, including biogenesis, modification, and degradation of tRNA, rRNA, mRNA, and other RNAs, as well as GTPase and ATPase activities. In a similar approach, this collection of RNA-binding domains (RBDs) has recently been used to generate an atlas of RBPs in humans [31]. Our HMM search identified 924 *P. falciparum* proteins that contain RBDs. This list of candidate RBPs was manually completed by including 64 proteins lacking an RBD, but that have annotated RNA-binding activity according to the information available in PlasmoDB, resulting in a total of 988 RBP candidates, or 18.1 % of the total *P. falciparum* proteome.

**Fig. 1:**
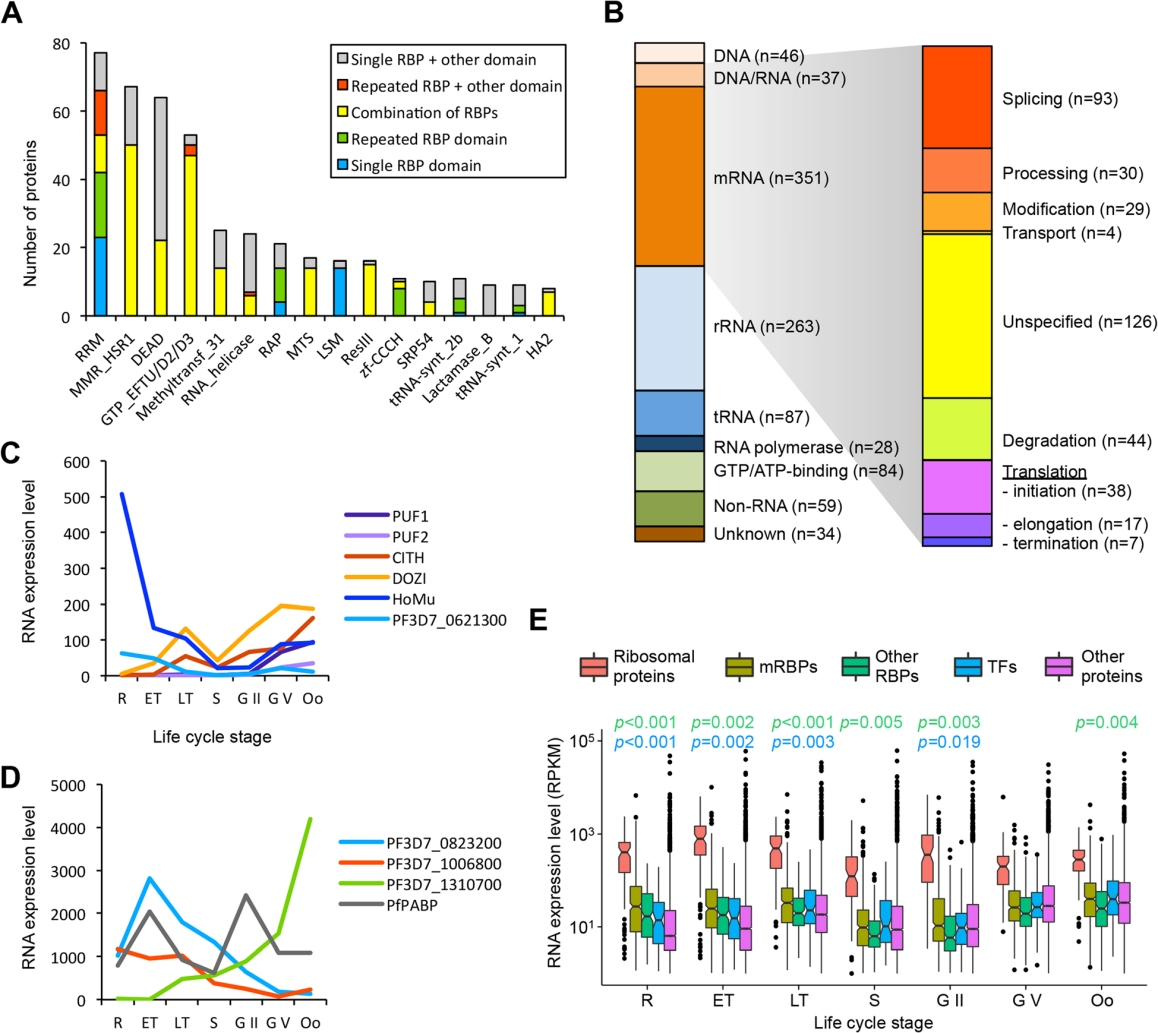
Overview of RNA-binding proteins in *P. falciparum*. **a** Characterization of RNA-binding domains (*RBDs*) that were found in eight or more proteins. **b** Classification of proteins containing one or more of 793 different RBDs based on the type of molecule that they most likely interact with, using information from existing annotations, functions of homologs in other species, and the nature of the RBD. Proteins that are known or predicted to interact with mRNA were further categorized into functional groups. **c** Steady-state mRNA expression levels of several RNA-binding proteins *(RBPs)* known to be involved in post-transcriptional regulation during the transmission stages. Expression data were obtained from PlasmoDB [34]. **d** Steady-state mRNA expression levels of three putative RBPs during the asexual and sexual erythrocytic stages and the mosquito ookinete stage. The expression level of polyA-binding protein (*PABP*), which is ubiquitously present in the cell, is plotted as a reference. **e** Comparison of expression levels of mRNA-binding proteins to other classes of proteins during different stages of the parasite’s life cycle. Statistically significant differences in average expression levels between mRBPs and other RBPs or transcription factors are indicated by a *p* value in *green* or *blue*, respectively, at the top of the plot (Welch’s *t* test). *R* ring, *ET* early trophozoite, *LT* late trophozoite, *S* schizont, *G II* stage II (early) gametocytes, *G V* stage V (late) gametocytes, *Oo* ookinete

The most common RBDs among *P. falciparum* proteins were observed to be RRMs (discussed in detail by Reddy et al*.* [30]), which were found in 77 proteins, followed by the MMR_HSR1 GTPase domain (67 members), the DEAD box helicase domain (64 members, see also [30]), and the GTP-binding elongation factor domain family (GTP_EFTU, GTP_EFTU_D2, and GTP_EFTU_D3; 53 members). For the RBDs that are present in eight or more proteins, we determined the structural features of the RBP candidates. Many proteins with RRM domains contain multiple instances of these domains or are combined with other RBDs, providing increased sequence specificity and binding affinity to the RBP [32] (Fig. 1a and Additional file 2). In contrast, DEAD box helicase, RNA helicase, and several other domains were often found in combination with non-RNA-related Pfam domains. As an exception, most LSm proteins almost exclusively harbor a single LSm domain and no other Pfam domains, indicative of their highly specialized function in mRNA splicing and degradation [33]. The majority (205 out of 230 proteins; 89 %) of RBPs described by Reddy et al. are confirmed in this study. Our HHM search identified four additional RRM proteins and 13 additional DEAD/DEXD helicases, but did not validate all zinc finger proteins and KH domain-containing proteins listed by Reddy et al*.* (Additional file 3: Figure S1).

The RBP candidates were then categorized based on the type of molecule that they most likely interact with, using information from existing annotations, functions of homologs in other species, and the nature of the RBD (Fig. 1b and Additional file 2). Out of 988 RBP candidates, 737 proteins (13.5 % of the proteome) have known or predicted RNA-related functions, including interactions with messenger RNA (*n* = 351), ribosomal RNA (*n* = 263), and transfer RNA (*n* = 86). A total of 46 proteins are most likely to bind to DNA, while 37 proteins may interact with either DNA or RNA, or both. A further 84 proteins have GTP- or ATP-binding activity, while 93 proteins have either no known function or a non-RNA-related annotation. The candidate messenger RNA-binding proteins (mRBPs, *n* = 388 including proteins that interact with either DNA or RNA) were further subdivided into functional categories: splicing, processing, modification, transport, degradation, translation initiation, translation elongation, and translation termination (Fig. 1b and Additional file 2).

A large fraction of candidate mRBPs (*n* = 126, 35.0 %) has known or predicted RNA-binding activity, but does not fall into any of the functional categories mentioned above. Several of these mRBPs have well-documented roles in post-transcriptional gene regulation, such as PUF2, DOZI, and CITH. Accordingly, these genes are most highly expressed in gametocytes and ookinetes, although they are also detected at lower levels during the asexual stages (Fig. 1c). Interestingly, Homolog of Musashi (HoMu; PF3D7_0916700), which has also been implicated in translational repression in gametocytes [25], and mRNA-binding Pumilio-homology domain protein (PF3D7_0621300) are both most highly expressed early in the IDC (Fig. 1c), raising the possibility that similar mechanisms of translational control occur during the IDC. Many proteins are merely annotated as putative RBPs without a specified function and typically contain multiple RRM domains. Some of these putative RBPs are among the top 1 % in terms of RNA-Seq gene expression [34], suggesting that they play important roles in RNA metabolism. Examples are PF3D7_0823200 and PF3D7_1006800, which are highly expressed during the IDC, and putative RBP PF3D7_1310700, which is highly abundant in ookinetes (Fig. 1d). PF3D7_0823200 has homology with CELF proteins, although its RBD architecture is different from that of CELF proteins in other organisms [30, 35]. PF3D7_1006800 has homology to G-strand binding protein 2 (Gbp2p; suggested annotation PfSrrm5) in other organisms [30] and may thus be involved in export of spliced mRNAs from the nucleus to the cytoplasm. Overall, the gene expression data also show that in many stages of the parasite’s life cycle the average expression level of mRBPs is higher than for other RBPs (Fig. 1e). In addition, mRBPs are on average more highly expressed than general and specific transcription factors during most of the IDC and in early gametocytes, indicative of their importance for gene regulation.

### Comparative analysis of RNA-binding proteins in apicomplexan parasites and other eukaryotes

**Fig. 2:**
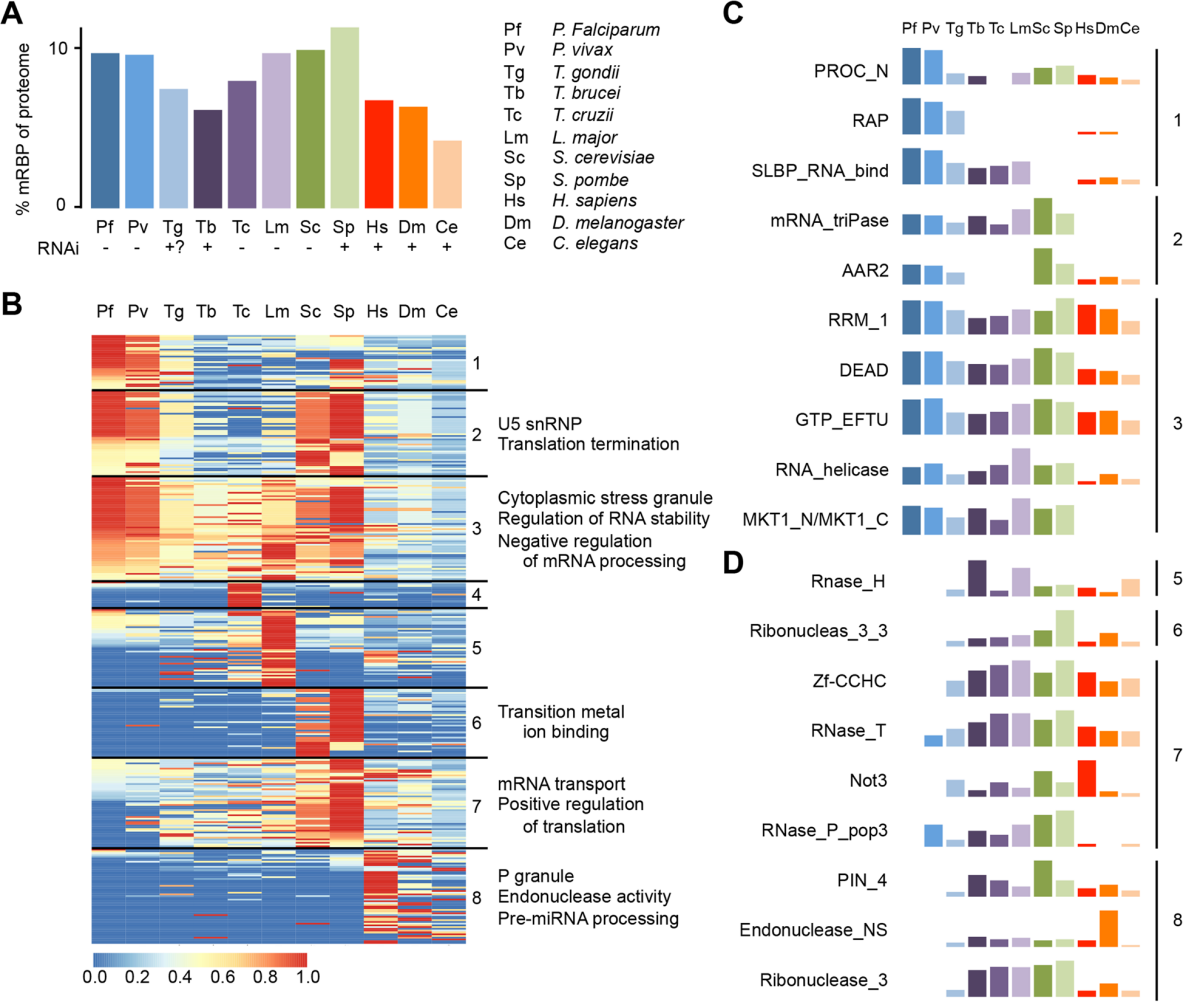
Relative abundance of mRNA-binding domains in *P. falciparum* in comparison to other eukaryotes. **a** Percentage of proteins in the full proteome that contain an mRNA-binding domain (*mRBD*). Abbreviations are listed on the *right*. The presence (+) or absence (–) of RNA interference machinery is indicated at the *bottom*. **b** *k*-means clustering of mRBDs based on relative abundance among all 11 organisms. The mRBD frequency in an organism was first normalized by proteome size and then scaled to the mRBD frequency in the organism with the highest relative abundance of that mRBD, which was given an arbitrary abundance value of 1. For clusters with unique enriched Gene Ontology (*GO*) terms associated with the Pfam domains (false discovery rate, *FDR* <0.01), a subset of these terms is shown on the *right*. See Additional file 4 for the full data set. **c** Selection of mRBDs that are relatively enriched in *P. falciparum* as compared to all or select groups of other eukaryotes. The cluster that these mRBDs fall into is indicated on the *right*. **d** Selection of mRBDs that are relatively depleted in *P. falciparum* as compared to all or select groups of other eukaryotes

To better understand the roles of RBPs in parasite biology, we next performed a comprehensive comparison of RBDs among a variety of organisms. Since the full set of 793 RBDs contains many domains typically found in proteins that interact with DNA, rRNA, or tRNA, this list was manually curated to include only known or putative mRNA-binding domains (mRBDs; *n* = 372). We then performed HMM searches on the proteomes of two additional apicomplexan parasites (*P. vivax* and *Toxoplasma gondii)*, three euglenid parasites (*Trypanosoma brucei*, *Trypanosoma cruzii*, and *Leishmania major*), two unicellular organisms (*Saccharomyces cerevisiae* and *Schizosaccharomyces pombe*), and three multicellular organisms (*Homo sapiens*, *Caenorhabditis elegans*, and *Drosophila melanogaster*) to find proteins that contain any of these mRBDs. To ensure that the HMM seeds were not biased towards apicomplexan organisms, we calculated the percentage of sequences in the HMM seeds derived from apicomplexa, euglenids, fungi, and metazoa. For only 7 out of 372 mRBDs, more than 25 % of the sequences in the HMM seed were derived from apicomplexan parasites, while a total of 178 and 165 mRBDs were biased towards fungi and metazoa, respectively (Additional file 4). *Plasmodium* species harbor a relatively high number of candidate mRBPs as compared to other organisms: 9.6 % of the full *P. falciparum* proteome and 9.5 % of the full *P. vivax* proteome contain mRBDs, similar to *Saccharomyces* species (9.8 % for *S. cerevisiae* and 11.2 % for *S. pombe*) and *L. major* (9.6 %) (Fig. 2a; see Additional file 4 for a complete overview of all RBDs in each organism). *T. cruzii* and *T. gondii* have intermediate levels of candidate mRBPs (7.4–7.9 %), while candidate mRBP levels in *T. brucei* and all three multicellular organisms analyzed here are much lower (range 4.2–6.7 %). Interestingly, these last four organisms have well-documented functional RNA interference (RNAi) machinery to regulate transcript abundance. With the exception of *S. pombe*, all organisms with high to intermediate levels of candidate mRBPs do not encode functional RNAi machinery, suggesting that a larger number of mRBPs may compensate for the lack of an RNAi pathway to control post-transcriptional gene expression. In agreement, the Piwi Argonaute Zwille (PAZ) and Piwi domains, found in proteins involved in RNAi, are absent or present at very low levels in the RNAi-negative organisms *P. falciparum*, *P. vivax, T. cruzii*, *L. major*, and *S. cerevisiae* (Additional file 3: Figure S2). It has to be noted that *T. gondii* does encode homologs of RNAi effector proteins Dicer, Argonaute, and RNA-dependent RNA polymerase that seem to produce miRNAs [36, 37], but experiments using double-stranded RNA for the downregulation of genes have not been uniformly successful [38].

To identify functional differences in RNA metabolism between organisms, the mRBDs were clustered based on the relative domain abundance among all 11 species (Fig. 2b). Each cluster was analyzed for enrichment of Gene Ontology (GO) terms associated with the Pfam domains. Clusters 1–3 contain mRBDs that are relatively abundant in *Plasmodium* species, of which the domains in cluster 1 are almost exclusively enriched in *Plasmodium*. The domains in cluster 2 are also relatively abundant in *Saccharomyces* spp., and the domains in cluster 3 are most highly abundant in *Plasmodium* and *Saccharomyces*, but are also present in other unicellular organisms (Fig. 2b). These clusters show enrichment for GO terms associated with splicing and RNA stability (Fig. 2b and Additional file 4), and they contain several common RBDs, such as RRMs [18], DEAD, and GTP_EFTU domains. In addition, cluster 1 harbors the PROC_N domain, which is found in pre-mRNA splicing factors of the PRO8 family, and cluster 2 contains the AAR2 domain, most likely also involved in pre-mRNA splicing (Fig. 2c). Examples of other domains that are enriched in *P. falciparum* as well as in other unicellular organisms are RNA_helicase, MKT1_C, MKT1_N, and mRNA_triPase. Finally, the RAP domain (RNA-binding domain abundant in Apicomplexans) is almost exclusively found in apicomplexan parasites [39] (Fig. 2c). Fifteen RAP proteins have been annotated in the *P. falciparum* genome, with six additional proteins containing a RAP domain identified here. The function of these proteins has yet to be discovered. A total of 90 proteins contain RBDs that are uniquely abundant in *Plasmodium* species (cluster 1). These proteins show various patterns of gene expression throughout the parasite life cycle, with some being most highly expressed during the IDC, while others are expressed in gametocytes or ookinetes (Additional file 3: Figure S3).

Clusters 4–8 contain mRBDs that are relatively depleted in *Plasmodium* species as compared to one or multiple other organisms. These clusters show enrichment for GO terms associated with mRNA transport and RNA degradation. In addition to several zinc finger domains, many RNase domains (RNase_T, Endonuclease_NS, Ribonuclease_3, RNase_H, Ribonucleas_3_3, RNase_P_pop3), the PIN_4 domain found in proteins involved in non-sense mediated decay, and the Not3 domain found in the CCR4-NOT degradation complex are found at low abundance or are completely absent in *Plasmodium* (Fig. 2d). This could point towards the existence of highly divergent and parasite-specific RNA degradation pathways. Interestingly, *T. cruzii* (cluster 4), *L. major* (cluster 5), *Saccharomyces* spp. (clusters 6 and 7), and *H. sapiens* (cluster 8) also show enrichment for several RBDs as compared to other organisms (Additional file 3: Figure S4), suggesting that these organisms have also developed certain species-specific RNA-related mechanisms.

### Experimental identification of RNA-binding proteins

**Fig. 3:**
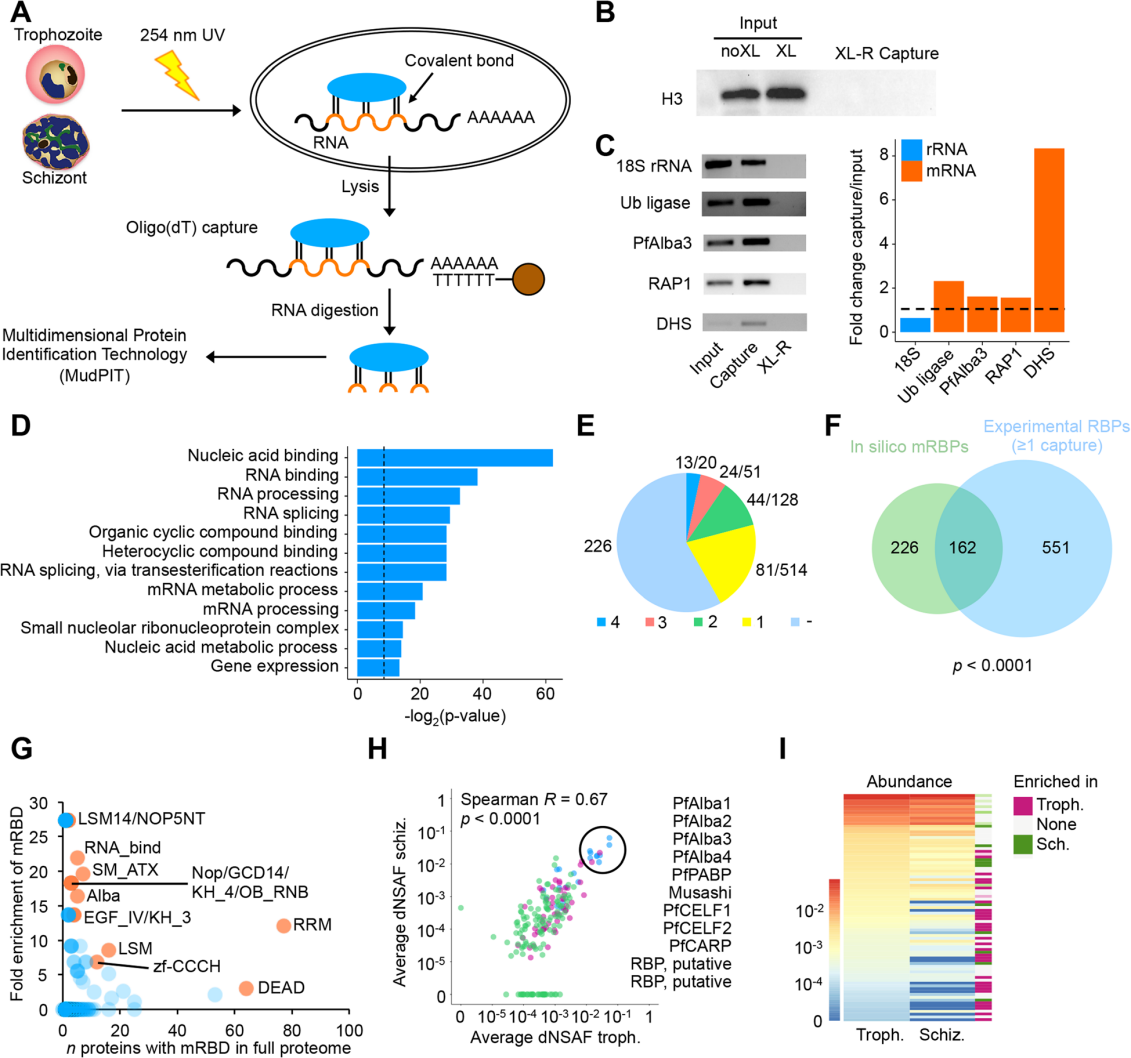
Capturing the mRNA interactome of *P. falciparum*. **a** Schematic overview of the experimental procedure, described in detail in the Methods section. **b** Non-RNA-interacting protein histone H3 is not detected in the capture sample, indicating that the capture methodology results in depletion of proteins that do not interact with poly-adenylated RNA. **c** Depletion of 18S ribosomal RNA and enrichment of mRNA transcripts in the capture sample, indicating that our approach to capture of poly-adenylated RNA is successful. No RNA was detected in the RNase-digested sample. **d** Selection of most highly enriched GO terms among the 199 proteins that were captured in two or more experiments. The *vertical dashed line* indicates a *p* value of 0.01. **e** Fraction of candidate RBPs identified by HMM search that were experimentally captured in one, two, three, or four individual experiments. The numbers to the *right* of the chart indicate the number of proteins with an RBD out of the total number of proteins captured experimentally. **f** Overlap between the mRBPs identified by HMM search (*n* = 388) and RBPs captured in one or more experiment (*n* = 713). The overlap between these two groups of proteins was assessed using the hypergeometric test. **g** Fold enrichment of RNA-binding domains in captured proteins as compared to the full proteome. mRBDs that are enriched among the mRNA interactome are indicated in *orange* (*p* < 0.05, FDR <5 %, hypergeometric test), while non-enriched and depleted mRBDs are shown in *blue*. **h** Correlation in abundance between 199 proteins captured at the trophozoite and schizont stages. Proteins are color-coded according to the number of experiments in which they were detected: in two (*green*), three (*magenta*), or four (*blue*) experiments. Highly abundant proteins are encircled and are listed on the right. **i** Average normalized spectral abundance factor (*dNSAF*) values of proteins captured at the trophozoite and schizont stages. Proteins absent at a particular stage are indicated in *dark blue*. The bar on the *right* indicates the stage at which a higher enrichment of the protein was observed

To validate our in silico identification of mRBPs and to determine which mRBPs may specifically be involved in mRNA metabolism during the IDC, we next performed an experiment designed to capture the global mRNA interactome, similar to published studies on yeast, worm, fly, and human cells [40–45]. Parasite cultures at the trophozoite or schizont stage were irradiated in duplicate with UV light at 254 nm to preserve interactions between proteins and nucleic acids. While the integrity of RNA is somewhat decreased as a result of UV treatment (Additional file 3: Figure S5), optimization experiments showed that long UV exposure times are necessary to obtain sufficient crosslinking between RNA and proteins (data not shown). After lysis under denaturing conditions, protein-mRNA complexes were isolated using oligo d(T) beads, followed by stringent washes with decreasing concentrations of salt and detergent (Fig. 3a). Under these conditions, we observed a depletion of the non-RNA interacting protein histone H3 (Fig. 3b) as well as 18S ribosomal RNA, and an enrichment of known mRNA targets of PfSR1 and PfAlba1, mRBPs that are involved in post-transcriptional regulation during the IDC (Fig. 3c). As a negative control, we included a sample that was first UV-crosslinked and then digested with RNases before the pull-down of protein-mRNA complexes (XL-R).

Proteins were eluted by digestion of mRNA with MNase and analyzed using multidimensional protein identification technology (MudPIT). By comparing capture samples to XL-R control samples, we identified a total of 199 proteins that are likely to directly or indirectly interact with mRNA (Additional file 5). These proteins were detected in at least two out of four independent samples that were analyzed (two replicates performed at the trophozoite and schizont stages, see Additional file 3: Figure S6A and Figure S6B) at ≥2-fold higher abundance in the capture sample as compared to the control sample. The captured mRBPs showed strong enrichment for GO terms associated with RNA homeostasis (Fig. 3d; see Additional file 6 for a full list of enriched GO terms). Another 514 proteins were enriched in only one capture experiment. A total of 81 candidate mRBPs detected in at least two independent capture experiments were identified in our computational search for mRBPs, while another 81 computationally identified mRBP candidates were captured in only one experiment. Together, the 162 candidate mRBPs that were captured at least once validate 41.8 % of the mRBP candidates identified in our HMM search (Fig. 3e) and represent 23 % of all experimentally detected candidate mRBPs (Fig. 3f).

Among the candidate mRBPs that were captured in at least two experiments (*n* = 199), the fraction of proteins interacting with mRNA or DNA/RNA was 6.5-fold enriched as compared to proteins that were depleted in the capture versus the RNase control samples (Additional file 3: Figure S6C). This enrichment was highest in the group of proteins that were identified with most confidence (i.e., enriched in all four experiments) and decreased with the number of experiments in which a protein was identified (Additional file 3: Figure S6C). In addition, the fraction of proteins without an mRBD was inversely correlated with the number of times a protein was identified (Additional file 3: Figure S6D). As compared to the proteome-wide abundance, significant enrichment was observed for the highly abundant RRM and DEAD domains, as well as for 14 less abundant domains, including LSm, RNA_bind, SM_ATX, zf-CCCH, KH_3, KH_4, and Alba (*p* < 0.05, 5 % FDR; Fig. 3g, Additional file 6).

The relative abundance of candidate mRBPs at the two different stages of the parasite IDC was strongly correlated (Spearman *R* = 0.67; Fig. 3h), resulting in a significant overlap between the mRNA-bound proteomes at the trophozoite and schizont stages (*n* = 155, *p* < 0.0001; Additional file 3: Figure S6E). Furthermore, mRBPs with higher relative abundance levels were more likely to be detected in three or more experiments (Fig. 3h). Interestingly, five out of 11 candidate mRBPs that were detected at relatively high abundance (dNSAF >0.01) showed a higher enrichment at the schizont stage than the trophozoite stage, including the four PfAlba proteins (Fig. 3i and Additional file 5), while proteins that show selective enrichment at the trophozoite stage are much less abundant (dNSAF <0.003). These results may indicate that the function of these highly abundant proteins may be particularly important at the schizont stage.

To define a stringent set of mRBPs active in the asexual stages of *P. falciparum*, we applied a conservative filter for differential protein abundance in the capture versus control data using the combined spectral counts from all four experiments (see Methods). In addition, we compared the spectral counts in our capture experiments with spectral counts from an existing *P. falciparum* mass spectrometry data set [46] to filter out any highly abundant proteins that could be contaminants. A total of 64 proteins meet both of these criteria (Additional file 5), of which the top 20 mRBPs detected with high confidence are listed in Table 1. Among these 64 mRBPs are many of the known translational regulators in *P. falciparum*: PfAlba1, PfCELF2 (formerly annotated as Bruno or HoBo), Musashi (HoMu), and CITH, as well as PfAlba2, 3, and 4, PfCELF1, and DOZI (Additional file 5). PfCELF1 is a recently identified member of the Bruno/CELF protein family with verified RNA-binding activity in *P. falciparum* [35]. DOZI has a known role in translational control in gametocytes. Together with a recent study showing a granular pattern of DOZI in the cytoplasm of asexual stage parasites [47], our results suggest that DOZI may also be involved in post-transcriptional mechanisms similar to those in gametocytes during the IDC. In addition, the list includes the highly expressed (top 1 % at the ring stage [34]) putative exporter of spliced mRNA PF3D7_1006800, CELF-like protein PF3D7_0823200, and another protein with CELF homology (PF3D7_1236100, clustered-asparagine-rich protein [30]), and further includes eight putative RBPs, one RAP protein, and 16 proteins with unknown function (Additional file 5). Finally, known mRBPs PUF1, PfSR1, and PfCAF1 were detected in one capture experiment but did not pass the stringent filters.

**Table 1:** Candidate mRNA-binding proteins captured with high confidence in trophozoite- and schizont-stage parasites

Locus	Description	Average spectra detected	Fold enrichment^a^				Pfam RNA-binding domain	QSPEC *p* value^b^	Chi-square *p *value^c^
			Troph. 1	Troph. 2	Schizont 1	Schizont 2			
PF3D7_1006800	RBP, putative	284	10.3	23.7	13.2	6.2	RRM	0	8.6E-144
PF3D7_1409800	PfCELF2 (formerly Bruno)	237	4.7	7.4	8.1	6.0	RRM, RNA_bind	0	2.3E-126
PF3D7_0916700	Musashi (HoMu)	187	11.4	5.5	37.0	4.0	RRM	0	1.7E-95
PF3D7_1359400	PfCELF1	176	4.8	11.7	8.5	9.9	RRM, RNA_bind	0	2.3E-85
PF3D7_0929200	RBP, putative	179	6.7	3.5	5.7	1.4	RRM	0	2.8E-74
PF3D7_1236100	PfCARP	151	6.9	1.9	13.0	2.8	RRM, RNA_bind	0	5.4E-52
PF3D7_0105200	RAP protein, putative	53	2.8	2.8	24.5	13.2	RAP	0	7.9E-26
PF3D7_1346300	PfAlba2	92	30.7	2.6	63.7	53.0	Alba	0	8.6E-26
PF3D7_1021900	Conserved protein	113	2.1	2.0	2.6	3.9	RNA_GG_bind	0	9.7E-25
PF3D7_0823200	RBP, putative	112	1.8	1.9	5.4	1.5	RRM, RNA_bind, DUF1866	0	2.0E-24
PF3D7_1105800	Conserved protein	53	4.0	0.6	5.6	13.2	-	0	8.8E-23
PF3D7_1006200	PfAlba3	131	4.4	5.1	12.8	11.6	Alba	0	2.8E-18
PF3D7_0703500	Erythrocyte membrane-associated antigen	80	0.9	2.9	5.7	4.4	DEAD	0	4.4E-18
PF3D7_0629400	RBP, putative	36	Infinity	7.0	Infinity	0	RRM	0	1.1E-14
PF3D7_1107300	PfPAIP1	73	1.2	3.2	1.9	4.4	-	0	1.2E-13
PF3D7_1474900	CITH	31	3.1	6.0	4.8	13.2	LSM14, FDF, SM_ATX	0	4.0E-13
PF3D7_1118200	Heat shock protein 90	41	3.1	4.9	8.8	3.6	-	0	3.4E-09
PF3D7_0810600	RNA helicase, putative	52	3.6	2.9	4.2	0	DEAD	0	1.5E-07
PF3D7_1347500	PfAlba4	103	4.5	4.7	6.3	7.4	-	0	2.1E-06
PF3D7_1330800	Conserved protein	37	8.4	1.8	Infinity	0	RRM	0	6.8E-04

^a^Enrichment of proteins in mRNA-interactome capture sample as compared to the corresponding RNase-treated control sample

^b^Analysis of differential protein abundance in the capture versus control data using the combined spectral counts from all four experiments. Reported *p* values are FDR-corrected (5 % [80])

^c^Analysis of protein abundance in the capture data compared to an existing *P. falciparum* mass spectrometry data set [46] to assess the likelihood of contamination. Reported *p* values are FDR-corrected using the Benjamini-Hochberg method (5 %)

*Troph.* trophozoite

### RNA-binding proteins involved in translation

**Fig. 4:**
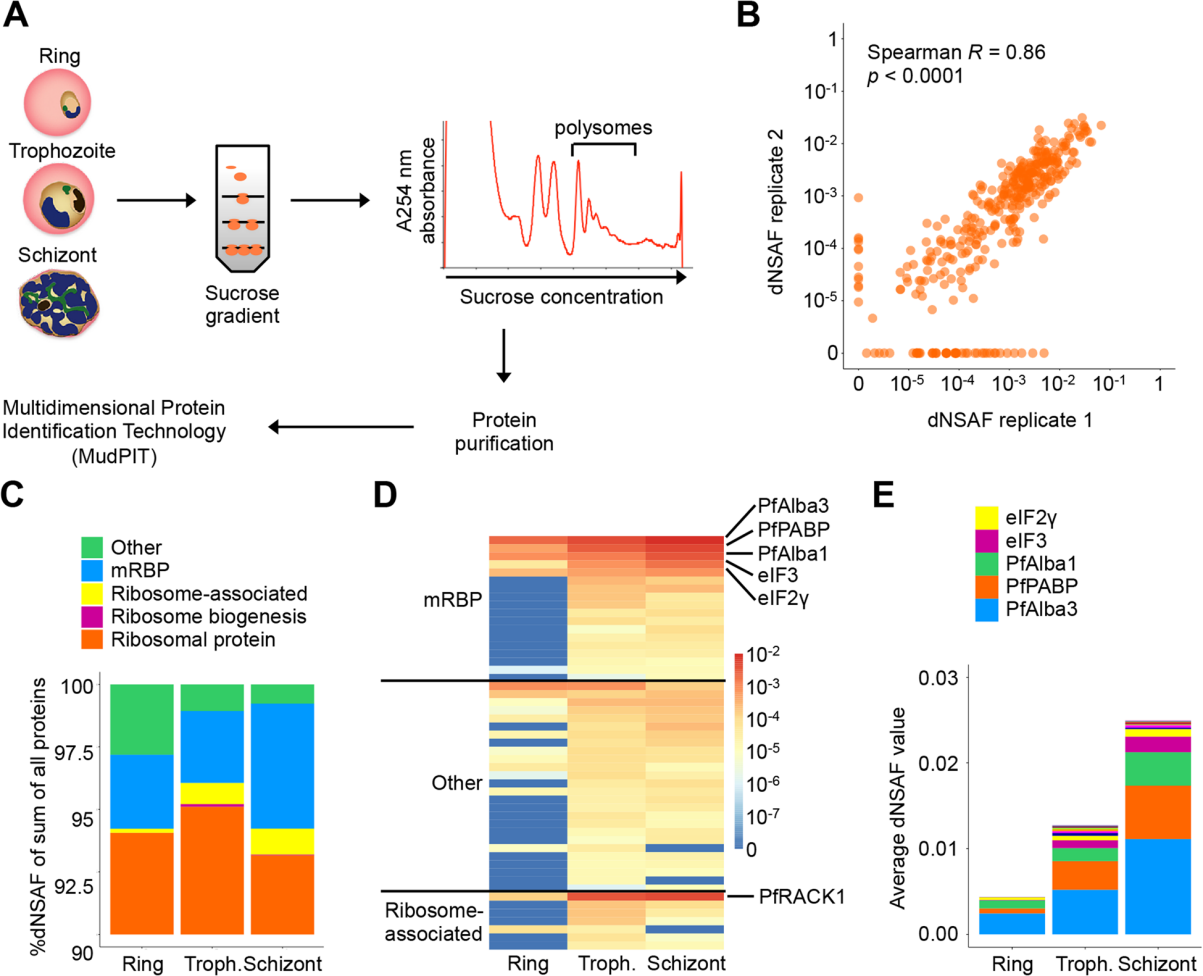
Identification of proteins associated with polysomes at the ring, trophozoite, and schizont stages of *P. falciparum*. **a** Schematic overview of the experimental procedure, described in detail in the Methods section. **b** Correlation in protein abundance between replicate experiments. **c** Relative frequencies of functional protein groups detected in polysome fractions. **d** Average normalized abundance (dNSAF values) of non-ribosomal proteins detected in polysome fractions at the main three IDC stages. Values are the average of two replicates. **e** Average normalized abundance of mRBDs at the different IDC stages

To identify RBPs that may play a regulatory role during the process of translation, we determined which proteins are associated with polysomes in the IDC. In duplicate experiments, ribosomes from lysates of red blood cells infected with ring-, trophozoite-, or schizont-stage parasites were separated over a sucrose gradient (Fig. 4a). Proteins present in the polysomal fractions were subsequently analyzed using MudPIT, yielding a total of 126 proteins that were detected with ≥2-fold higher abundance in polysome fractions than in cytoplasmic fractions of parasites of the same stage and that were detected in duplicate experiments. None of these proteins were detected after disruption of polysomes by EDTA treatment (data not shown), suggesting that they are truly associated with polysomes and not merely co-sedimenting in polysome fractions. Overall, replicate experiments showed a strong correlation for detected protein abundance (Spearman *R* = 0.86; Fig. 4b). We also observed overlaps between 45–88 % for non-ribosomal proteins detected at the same IDC stage in replicate experiments (Additional file 3: Figure S7).

Between 93.7–95.8 % of *P. falciparum* proteins identified in the polysomal fractions were ribosomal proteins (*n* = 75; Fig. 4c), which were highly enriched compared to cytoplasmic fractions (on average 13.6-fold enrichment). The largest fraction of non-ribosomal proteins associated with polysomes consisted of known or predicted mRNA-binding proteins (2.7–4.8 %, Fig. 4c). Several proteins known to be involved in translation, such as subunits of eukaryotic initiation factors (eIF) 2 and 3 and polyadenylate-binding protein (PfPABP), were highly abundant (dNSAF >10^-4^), as well as the DNA/RNA-binding proteins PfAlba1 and PfAlba3 (Table 2). Other known or predicted mRBPs were observed at lower abundance, including PfCAF1, the RRM domain-containing putative RNA-binding protein PF3D7_0629400, and CCCH zinc finger proteins PF3D7_0525000 and PF3D7_0906600 (Table 2). CCCH-type zinc finger proteins are commonly involved in regulating mRNA decay and translation rates and are relatively abundant in *P*. *falciparum* [11]. Another CAF protein (PfCAF40; PF3D7_0507600) and a NOT family member (both part of the CCR4-NOT complex) were also identified, as well as putative ribonuclease PF3D7_0615400. Finally, we detected several proteins known to be associated with ribosomes or involved in ribosome biogenesis, as well as 19 conserved *Plasmodium* proteins with unknown function, of which only one contained an RBD (IF4E, found in the eukaryotic translation initiation factor 4E family; Additional file 7). Interestingly, the ribosome-associated protein receptor for activated C-kinase 1 (PfRACK1; PF3D7_0826700) was detected in five out of six polysome preparations with relatively high abundance (Fig. 4d), while it was not detected in cytoplasmic fractions. The average normalized abundance ratio between PfRACK1 and ribosomal proteins was 0.65:1 across all six experiments. Recent cryo-electron microscopy studies reported the unusual absence of PfRACK1 from the *P. falciparum* ribosome and suggested that this protein may be largely ribosome-unbound in the parasite [48, 49]. However, our results indicate that PfRACK1 is mostly associated with ribosomes, although its association may easily be disrupted by experimental procedures.

**Table 2:** mRNA-binding proteins associated with polysome fractions during the IDC

Locus	Description	Pfam RNA-binding domain
PF3D7_0811300	PfCAF1	CAF1
PF3D7_0507600	PfCAF40	-
PF3D7_0111800	Conserved Plasmodium protein, unknown function	IF4E
PF3D7_0814200	PfAlba1	Alba, Rpp20
PF3D7_1006200	PfAlba3	Alba
PF3D7_0728000	eIF2 alpha subunit	EIF_2_alpha, S1, EXOSC1
PF3D7_1410600	eIF2 gamma subunit	GTP_EFTU, eIF2_C, GTP_EFTU_D2, MMR_HSR1
PF3D7_0716800	eIF3 subunit I	-
PF3D7_0517700	eIF3 subunit B	eIF2A, RRM
PF3D7_1417200	NOT family protein, putative	Not1
PF3D7_1107300	PfPAIP1	-
PF3D7_1224300	PfPABP	RRM, PABP
PF3D7_0615400	Ribonuclease, putative	RNB, OB_RNB
PF3D7_0629400	RNA-binding protein, putative	RRM
PF3D7_1129400	RNA methyltransferase, putative	Nol1_Nop2_Fmu, Methytransf_31, FtsJ
PF3D7_0503300	Ser/Arg-rich splicing factor, putative	RRM
PF3D7_0525000	CCCH zinc finger protein, putative	zf_CCCH
PF3D7_0906600	CCCH zinc finger protein, putative	-

Overall, only a small number of RBPs were found to be associated with polysomes. This could reflect restricted regulation of gene expression during the process of translation, but it could also be the result of limited sensitivity of the mass spectrometry analysis. Most polysome-associated proteins were detected at the trophozoite and schizont stages (Fig. 4d), consistent with higher levels of translation at these IDC stages. Of the mRBPs, only PfAlba1, PfAlba3, PfPBAP, and eIF2γ were present constitutively (Fig. 4d and Additional file 3: Figure S8). On average, the abundance of RBPs in the polysome fractions was highest at the schizont stage (Fig. 4e), suggesting that many of these proteins are involved in timing the moment of translation of proteins expressed later in the IDC, similar to PfAlba1 and PfCAF1 [27, 28].

## Discussion

Evidence is accumulating that post-transcriptional mechanisms play important roles in regulating gene expression during various stages of *P. falciparum*‘s life cycle, including the intraerythrocytic developmental cycle [14, 15, 24, 25, 27, 28]. To better understand these regulatory processes, it is essential to know which RNA-binding proteins (RBPs) are involved in RNA metabolism in the parasite. While many RBPs are annotated as such in the well-curated *Plasmodium* database (PlasmoDB, www.plasmodb.org), a systematic overview of *P. falciparum* RBPs has been lacking. The data presented here give the most complete overview of RBPs in the malaria parasite *P. falciparum* to date.

By searching the *P. falciparum* proteome using a large array of Pfam domains involved in all aspects of RNA metabolism, we have attempted to capture every single RBP. Since the *P. falciparum* genome is relatively distant from that of more classical model organisms, we used relatively non-stringent parameters for the HMM search to increase our chances of identifying RBPs with weakly homologous RNA-binding domains (RBDs). For the small number of Pfam domains for which it was readily apparent that our threshold for inclusion resulted in false positives, we used more stringent inclusion criteria (see Methods). In addition, we have attempted to account for false positive hits by narrowing down our initial broad search to proteins that specifically interact with mRNA, using information from the current genome annotation. Our results are in good agreement with a recent bioinformatics analysis that focused on a limited set of RBPs with relatively common or well-characterized RBDs [30] and catalogs many additional RBPs that to our knowledge have not previously been indexed in this fashion. Conflicts in the lists of RBPs retrieved in these two studies could be the result of different search approaches: Reddy et al. [30] used HMM profiles built from RBPs that were identified in a text-based search of PlasmoDB, while this study scanned the genome using Pfam domains. Further experimental work will be necessary to validate the function of these computationally identified RBPs in RNA biology.

In an unbiased comparison with other eukaryotic organisms, we observed that *P. falciparum* is among the species that encode a relatively large number of mRNA-binding proteins (mRBPs). Some of these mRBPs contain a domain (e.g., RAP) that is found almost exclusively in apicomplexan parasites (Fig. 2b). While the exact function of these proteins will have to be validated at the molecular level, this finding in all likelihood reflects the importance of RNA metabolism for parasite biology and is in agreement with the presumed role of post-transcriptional mechanisms of gene regulation, in particular at the level of mRNA stability and degradation.

The coordinated post-transcriptional control of ookinete-specific transcripts in the female gametocyte has been well documented and involves the stabilization of transcripts in ribonucleoprotein complexes with regulators such as DOZI and CITH [23–25]. Our data strongly suggest that these regulators also play important roles during the IDC. In addition, other post-transcriptional regulators, including PfSR1 and PfAlba1, have recently been identified to control subsets of genes during the IDC [27, 28]. This coordinated regulation of functionally related genes is analogous to gene regulation in trypanosomes. Transcription in *T. brucei* and *T. cruzii* is polycistronic, and extensive regulation of gene expression occurs at the post-transcriptional level (reviewed in [50]). Functionally related genes show coordinated expression throughout the cell cycle and during differentiation into other parasitic stages [51]. These regulons are presumably controlled by RBPs that recognize regulatory elements in the untranslated regions of mRNA, and the identification of factors involved in these regulatory processes has brought rapid progress to understanding trypanosome biology (see reviews [52–54] and references therein). Further validation and characterization of the RBPs identified in this study is likely to bring similar advances to the malaria field. The disruption of genes encoding RBPs relatively often confers a phenotype of severely attenuated growth [55]. A recent antimalarial drug screen identified the compound DDD107498 as an inhibitor of *P. falciparum* translation elongation factor 2 (eEF2) with activity against multiple stages of the parasite life cycle [56]. This discovery shows the importance of translation for parasite survival and indicates that proteins involved in RNA metabolism, and in particular in post-transcriptional and translational gene regulation, may provide excellent targets for novel antimalarial drugs.

Out of a total of 388 proteins that contain an mRNA-binding domain and are thus likely to be involved in mRNA metabolism in the parasite, 162 proteins (42 %) were experimentally confirmed in our mRNA-interactome capture experiments. Proteins that were not identified in our mRNA-interactome capture experiments may act during other stages of the parasite’s complex life cycle, may be only transiently expressed, or may have low expression levels and are therefore difficult to detect by mass spectrometry. Mass spectrometry is known to be biased towards highly abundant proteins. Even though the correlation between spectral counts in our data and RNA-Seq expression levels is weak (Pearson *R* = 0.32 and 0.24 at the trophozoite and schizont stages, respectively), there is indeed a trend towards a more frequent detection of proteins with higher expression levels (Additional file 3: Figure S9). In addition, we may have missed the detection of mRBPs as a result of our experimental approach for the mRNA-interactome capture experiments. In this study, we used conventional UV-crosslinking (cCL) to induce covalent bonds between RNA and interacting proteins. An alternative strategy, called photo-activatable crosslinking or PAR-CL, is to supply thiol-labeled uridine to cells, which is then incorporated into nascent RNA and can efficiently be crosslinked to protein by 365 nm UV light irradiation [41, 44]. For *P. falciparum*, this strategy requires the use of a transgenic parasite strain that is capable of salvaging pyrimidines from its environment. Despite differences in crosslinking chemistries, the overlap in mRBPs captured from human cells using cCL or PAR-CL is large (two-thirds or more) [41, 44]. Nevertheless, it would be interesting to compare the results of both strategies to explore the full mRNA-bound proteome of *P. falciparum*. Finally, as a result of RNA degradation due to UV exposure and subsequent poly-A selection, our capture data may be biased towards proteins that bind at the 3’ end of mRNA transcripts and be less likely to detect proteins that bind at the 5’ end.

It is known that UV-crosslinking experiments often yield many false positives as a result of background from proteins that are covalently crosslinked to RNA in a non-specific manner. Recent efforts have been made to characterize and correct for this type of background noise in PAR-CL data [57] and to optimize the experimental procedures for CLIP-Seq experiments [58]. In addition, we noticed that UV-crosslinking of *P. falciparum* parasites results in increased non-specific protein pull-down during the capture procedure, which can increase the number of false positives. We have controlled for this phenomenon by performing UV-crosslinking followed by RNase digestion of the control sample, instead of using a non-UV-crosslinked control as is more common for these types of experiment [40, 41]. To provide additional confidence and supply a means to filter false positives from our data set, we have performed two additional stringent statistical tests based on (1) protein enrichment in capture versus control data and (2) removal of highly abundant proteins that could be contaminants. Applying these two additional filters resulted in a final list of 64 mRBPs experimentally captured with high confidence, which makes an excellent starting point for further exploration of the *P. falciparum* mRNA-bound proteome. We stress that this is a very conservative list of mRBP candidates that may not include all true hits from our experimental capture approach.

Several of the proteins that were found to be essential for normal IDC development in the genetic screen by Balu et al*.* [55] were captured in our mRNA interactome, including PfCAF1 (enriched in one mRNA-interactome capture experiment) and the putative RNA-binding proteins PF3D7_1360100 (enriched in three capture experiments) and PF3D7_0812500 (enriched in one capture experiment; Additional file 5 ). These latter two proteins were also identified as important interacting partners, connecting interaction networks of proteins involved in RNA metabolism and protein folding and trafficking [59]. In addition, the results of our experimental strategies to validate the role of RBPs during the IDC suggest an important function of PfAlba1–4 proteins in post-transcriptional gene regulation in *P. falciparum*. All four PfAlba proteins were highly enriched in the mRNA-interactome capture experiment, and PfAlba1 and PfAlba3 were also found to be associated with polysomes. PfAlba1 is a known regulator of translation, in particular for genes involved in merozoite egress and invasion [28], although the exact mechanism by which the protein acts remains to be established. The function of the other PfAlba proteins in post-transcriptional regulation is less well determined. During the trophozoite and schizont stages, PfAlba1–4 are localized in granules that could represent ribonucleoprotein complexes [60], suggestive of a role in regulating mRNA stability and degradation. Additional mechanistic insight into how these proteins function is still missing and warrants further investigations into the role of these proteins in the parasite in post-transcriptional and translational gene regulation. Interestingly, the recent identification of two additional PfAlba proteins based on the presence of an Alba domain (PfAlba5 and PfAlba6) [30] was confirmed in our HMM search. Of these two new members of the Alba family, PfAlba6 shares only limited sequence identity with the other five PfAlba proteins. Neither PfAlba5 nor PfAlba6 were identified in our mRNA-interactome capture experiments and have relatively low expression levels during the IDC, gametocyte, and ookinete stages [34], suggesting that if these are indeed bona fide PfAlba proteins, they may function in other stages of the parasite’s life cycle.

Three of the polysome-associated proteins identified in this study can also be found in stress granules and P-bodies, including members of the CCR4-NOT complex (PfCAF1, PfCAF40, and PfNOTx), as well as eukaryotic translation initiation factors and the PfAlba proteins [25, 61, 62]. Such ribonucleoprotein complexes are involved in translational regulation and mRNA decay (reviewed in [63]) and have an established role in transcript stabilization in female gametocytes [24, 25]. However, the CCR4-NOT complex can also be associated with transcripts in the cytoplasm and during translation (reviewed in [64]) and is, for example, involved in translational repression of transcripts that cause ribosome stalling [65]. In addition, many of the other components of P-bodies or stress granules (such as DOZI and CITH in *P. falciparum*) were not detected. Thus, although we cannot completely eliminate the hypothesis that some RNA granules may have co-sedimented with the polysome fractions, the proteins that we identified here are more likely to be associated with polysomes than with other structures in the cell.

## Conclusion

This study presents the most complete resource of RNA-binding proteins in *P. falciparum* to date. We have computationally identified RNA-binding proteins based on the presence of RNA-binding domains and further classified these proteins into functional categories. Furthermore, we provide experimental evidence for the role of a subset of RBPs in mRNA homeostasis during the IDC, the stage responsible for disease in humans. The function of many RBPs is still unknown, and further characterization of RBPs important for parasite development is therefore likely to increase our understanding of parasite biology and to reveal excellent novel targets for drug discovery.

## Methods

### HMM search

Protein sequences were obtained from the following sources: PlasmoDB version 13.0 (*P. falciparum* strain 3D7), PlasmoDB version 24.0 (*P. vivax* strain Sal I), ToxoDB version 24.0 (*T. gondii* strain ME49), TriTrypDB version 24.0 (*T. brucei* strain TREU927, *T. cruzi* strain CL Brener Esmeraldo-like, and *L. major* strain Friedlin), Saccharomyces Genome Database (*S. cerevisiae* strain S288C genome assembly R64-2-1 [66]), PomBase (*S. pombe* downloaded on 25 June 2015), and Ensembl release 80 [67] (*H. sapiens* genome assembly GRCh38.p2, *C. elegans* genome assembly WBcel235, and *D. melanogaster* genome assembly BDGP6). Protein sequences were searched for the presence of Pfam HMM profiles (Pfam version 27.0 [68]) using the function hmmscan of the HMMER software package [69] (version 3.1b1, release May 2013). Proteins containing any of 793 Pfam RNA-binding protein (RBP) domains [31] (for *P. falciparum* only) or 372 mRBP domains (all other organisms) with an E-value below 0.01 were included in subsequent analyses (see Additional file 1). Six of the RBP domains used by Gerstberger et al*.* [31] are no longer listed in the Pfam database.

The resulting list of RBPs was manually curated. For Pfam domain eIF2A (PF08662), an E-value cutoff of 0.01 resulted in false positives in multiple organisms, in particular from proteins with a WD domain that are typically involved in signal transduction, transcription, and cell cycle control (Additional file 3: Figure S10). Therefore, a more stringent cutoff of 1E-15 was used for this domain. The Bud13 (PF09736) domain yielded seven false positives in *P. falciparum* (all members of exported protein family 3) with E-values between 0.01 and 0.001, while the true hit (PF3D7_1246600) obtained an E-value of 2.10E-43. The cutoff for this Pfam domain was therefore lowered to 0.001. Similar discrepancies between HMM results and gene annotations were not observed for any of the other Pfam domains. Applying an E-value cutoff of 0.001 to all Pfam domains would result in the exclusion of a total of 63 proteins, including 16 known RBPs and 19 conserved proteins with unknown function, and was therefore considered too stringent.

Proteins containing DNA-binding zinc finger domains (KRAB, SCAN, BTB, zf-met, zf-C2H2, and zf-C2H2_jaz) were removed from the data set. For proteins with multiple isoforms, the isoform with the highest number of Pfam domains was selected. If multiple isoforms had equal numbers of domains, the longest isoform was chosen. For *P. falciparum*, genes with a gene annotation containing “RNA,” “ribosomal,” or “translation” were manually added to the list of candidate RBPs (*n* = 40), as well as genes with the Gene Ontology (GO) terms “RNA binding” (GO:0003723), “rRNA binding” (GO:0019843), or “tRNA binding” (GO:0000049) among the first three GO terms listed for that gene (*n* = 24). Nine of the genes added based on GO annotation had an RNA-related gene description (for example, PF3D7_0621900, signal recognition particle subunit SRP68), while the other 15 were genes with unknown function. It is possible that the current gene annotation is incorrect and that these genes were mislabeled as “RNA binding.” Out of all 64 manually added genes, nine genes contained weak to very weak RBDs (median E-value = 0.041), of which five were strongly related to the gene annotation. Diversification of these genes in *P. falciparum* may have precluded identification of these domains in our HMM search.

The type of molecule that the candidate RBP interacts with was determined based on existing annotations and known functions of homologs in other species. If this information was not available, the type of molecule was predicted based on the nature of the RNA-binding domain (RBD). Proteins for which no information was available were categorized as “non-RNA.”

To calculate the percentage of sequences from groups of organisms in the HMM seed, the HMM seed file (Pfam version 27.0) was downloaded, filtered for the 372 mRNA-binding domains used in this study, and parsed for UniProt accession numbers. The source organism of each sequence was then retrieved using the retrieve/ID mapping tool on the UniProt website (http://www.uniprot.org/uploadlists/) and matched to the corresponding Pfam domain. For each domain, the percentage of sequences derived from each group at the third level of the taxonomic lineage was determined.

In each organism, a variety of proteins that are unlikely to be involved in RNA metabolism were identified in the HMM search. However, manually curating these protein lists would introduce a bias, since not all genomes have been annotated to the same extent. Therefore, to make a fair comparison between organisms, we included all mRBD-containing proteins in our subsequent analysis, irrespective of their annotation. To correct for differences in genome size, RBD abundance was expressed as the number of RBDs per 10,000 genes. Pfam domains that were present in at least one out of 11 organisms (*n* = 353) were clustered based on their relative abundance across organisms using the *k*-means clustering algorithm with a maximum of 1000 iterations in R v2.7.0 [70]. Determination of the optimal number of clusters (*n* = 8) was guided by the percentage of variance that was captured by the clusters. We selected the smallest number of clusters for which an increase in the number of clusters did not capture at least an additional 2 % of the variance (expressed as a within-group sum of squares). A heatmap of clustered Pfam domain abundance was generated using the pheatmap package in R v2.7.0. Domain-centric GO analysis of Pfam domain clusters was performed using the web-based version of dcGO (http://supfam.org/SUPERFAMILY/cgi-bin/dcenrichment.cgi) [71], with a collapsed subset of GO terms and a false discovery rate (FDR) <0.01.

### Gene expression analysis

PlasmoDB has preprocessed all available RNA-Seq expression data sets using standardized pipelines to ensure comparability between data sets. Normalized RPKM gene expression data from seven stages of the *P. falciparum* life cycle [34] were downloaded from PlasmoDB v26. Notched box plots of expression values for various groups of proteins were generated using the ggplot2 package in R v2.7.0. Differences in expression levels between groups of proteins were assessed using the Welch’s unequal variances *t* test. The heatmap of gene expression patterns for candidate RBPs with *Plasmodium*-specific RBDs was generated from z-scored RPKM values using the pheatmap package in R v2.7.0.

### Parasite cultures

The *P. falciparum* strain 3D7 was cultured in human O^+^ erythrocytes at 5 % hematocrit as previously described [72]. Cultures were synchronized twice at ring stage with 5 % D-sorbitol treatments performed 8 h apart [73]. Cultures (8 % parasitemia in 5 % hematocrit in a total volume of 25 ml) were harvested 48 h after the first sorbitol treatment (ring stage) and then 18 h (trophozoite stage) and 36 h thereafter (schizont stage).

### Isolation of mRNA interactome

Parasites from mixed trophozoite and schizont cultures were extracted by saponin lysis of erythrocytes and were crosslinked on ice by 254 nm UV light for a total of 1200 J/cm^2^ with two 2-min breaks with gentle mixing. The parasites were then washed in phosphate-buffered saline (PBS) and lysed in a lysis/binding buffer containing 100 mM Tris-HCl pH 7.5, 500 mM LiCl, 1 mM EDTA, 0.5 % LiDS, and 5 mM dithiothreitol (DTT). Negative control samples were lysed in lysis/binding buffer without EDTA and treated with 400 μg of RNase A (Life Technologies) and 10,000 units of RNase T1 (Ambion) for 30 min at 37 °C, followed by the addition of EDTA to a final concentration of 1 mM. Samples were then allowed to bind to magnetic oligo d(T)_25_ beads (New England Biolabs) by incubating at room temperature for 1 h with continuous mixing. The beads were washed twice in wash buffer I (20 mM Tris-HCl pH 7.5, 500 mM LiCl, 1 mM EDTA, 0.1 % LiDS, and 5 mM DTT), twice in wash buffer II (20 mM Tris-HCl pH 7.5, 500 mM LiCl, and 1 mM EDTA), and once in low-salt buffer (20 mM Tris-HCl pH 7.5, 200 mM LiCl, and 1 mM EDTA). Proteins were eluted in elution buffer (10 mM Tris-HCl pH 7.5, 2 mM CaCl_2_, and 50 units of MNase) by incubation for 30 min at 37 °C, followed by the addition of Laemmli buffer and a 10-min incubation at 98 °C. To control for the integrity of RNA after crosslinking, the total RNA was extracted from non-crosslinked and crosslinked parasites using TRIzol LS Reagent (Life Technologies) according to the manufacturer’s instructions. RNA (1 μg) was visualized on 1 % agarose gel stained with ethidium bromide.

### RT-PCR

Non-UV-crosslinked parasites and UV-crosslinked parasites were resuspended in lysis/binding buffer without EDTA and lysed by needle shearing. Parasite lysates and RNA capture samples were treated with Proteinase K for 30 min at 45 °C. RNA was isolated using the RNeasy Kit (Qiagen) and treated twice with 4 U DNase I (Life Technologies) per 10 μg of RNA for 30 min at 37 °C. DNase I was inactivated by the addition of EDTA to a final concentration of 1 mM. DNase-treated RNA was mixed with 0.1 μg of random hexamers, 0.6 μg of oligo(dT) 20, and 2 μl 10 mM dNTP mix (Life Technologies) in a total volume of 10 μl, incubated for 10 min at 70 °C, and then chilled on ice for 5 min. This mixture was added to a solution containing 4 μl 10X RT buffer, 8 μl 20 mM MgCl_2_, 4 μl 0.1 M DTT, 2 μl 20 U/μl RNaseOUT, and 1 μl 200 U/μl SuperScript III Reverse Transcriptase (all from Life Technologies). First-strand cDNA was synthesized by incubating the sample for 10 min at 25 °C, 50 min at 50 °C, and finally 5 min at 85 °C. DNA was amplified using KAPA HiFi DNA Polymerase by incubating for 5 min at 95 °C, followed by 30 cycles of 30 s at 98 °C, 30 s at 58 °C, and 30 s at 62 °C, using the following primers: PF3D7_0725600 (18S rRNA), F: 5′-GAATTGACGGAAGGGCACC, R: 5′-CTTCCTTGTGTTAGACACAC; PF3D7_0826100 (E3 ubiquitin-protein ligase), F: 5′-CAGCATATACTGATGCTAAAG, R: 5′-AATGGTAGGACTATAGTATTATT; PF3D7_1412600 (deoxyhypusine synthase), F: 5′-GATCAATGTGACATGTATTATC, R: 5′-CTCCGAGAATAATAATACCAG; PF3D7_1410400 (RAP1), F: 5′-CATCAGCTGCTGCAATTCT, R: 5′-CGAAGCACTTCTCTTTGAGG; and PF3D7_1006200 (PfAlba3), F: 5′-GGATGTTTACAGGAAATGAAGAGA, R: 5′-GTTTGCTACAAAATCTGGGTG. The absence of genomic DNA contamination was validated using a primer set targeting PfAlba3 (PF3D7_1006200) from inside exon 1 to within exon 2.

### Western blot analysis

Non-UV-crosslinked and UV-crosslinked parasite lysates were treated with RNaseA and RNase T1 for 30 min at 37 °C, followed by DNase treatment for 10 min at 37 °C. Samples were centrifuged at 13,000 × g for 2 min. The lysate supernatants and capture samples were then loaded on an Any-kD SDS-PAGE gel (Bio-Rad) and run for 42 min at 150 V. Proteins were transferred to a PVDF membrane for 40 min at 16 V, stained using Anti-Histone H3 antibody (Abcam ab1791, 1:3,000) and Goat Anti-Rabbit IgG HRP Conjugate (Bio-Rad, 1:25,000), and visualized using the Bio-Rad ChemiDoc MP Gel Imager.

### Polysome isolations

Polysomes were isolated in duplicate from *P. falciparum* cultures at the ring, trophozoite, and schizont stages according to a recently published protocol with minor modifications [74]. Cycloheximide was added to parasite-infected red blood cell cultures to a final concentration of 200 μM, followed by 10 min incubation at 37 °C. Erythrocytes were then pelleted (4 min at 660 × g) and washed twice in PBS containing 200 μM cycloheximide. After the last wash, the pellets were kept on ice and were subsequently lysed by adding 2.2 volumes of lysis buffer (1 % [v/v] Igepal CA-360 [Sigma-Aldrich] and 0.5 % [w/v] sodium deoxycholate in polysome buffer [400 mM potassium acetate, 25 mM potassium HEPES pH 7.2, 15 mM magnesium acetate, 200 μM cycloheximide, 1 mM DTT, and 1 mM AEBSF]). After 10 min incubation on ice, the lysates were centrifuged for 10 min at 20,000 × g at 4 °C. The clarified lysates were then loaded on top of a sucrose cushion (1 M sucrose in polysome buffer) to concentrate the ribosomes. For large culture volumes, 20 ml lysate was loaded on top of 6 ml of sucrose cushion in 26 ml polycarbonate ultracentrifuge tubes and then centrifuged for 3 h at 50,000 rpm at 4 °C in a Type 70 Ti rotor (Beckman Coulter, Brea, CA, USA). For small culture volumes, 4 ml lysate was loaded atop 1.25 ml of sucrose cushion in 5 ml polyallomer ultracentrifuge tubes and then centrifuged for 123 min at 50,000 rpm at 4 °C in an SW 55 Ti rotor (Beckman Coulter). Ribosome pellets were resuspended in polysome buffer, incubated for at least 30 min at 4 °C to allow complete ribosome resuspension, and centrifuged for 10 min at 12,000 × g at 4 °C. The ribosome suspension was layered on top of a 4.5-ml continuous linear 15–60 % sucrose [w/v] gradient in polysome buffer and centrifuged for 1.5 h at 50,000 rpm at 4 °C in an SW 55 Ti rotor. Fractions of 400 μl were collected using an UA-5 UV Detector and Model 185 Gradient Fractionator (ISCO, Lincoln, NE, USA). To control for co-sedimentation of proteins in polysome fractions, polysomes were disrupted by resuspension of the ribosome pellets in buffer containing 25 mM EDTA.

For the isolation of cytoplasmic fractions, synchronized parasite cultures were lysed by incubation in 0.15 % saponin for 10 min on ice. Parasites were centrifuged at 3234 × g for 10 min at 4 °C and washed three times with PBS. After the last wash, the parasites were resuspended in PBS, transferred to a microcentrifuge tube, and centrifuged for 5 min at 2500 × g at 4 °C. Subsequently, the parasite pellet was resuspended in 1.5X volume of cytoplasmic lysis buffer (0.65 % Igepal CA-360, 10 mM Tris-HCl pH 7.5, 150 mM NaCl, 1 mM EDTA, 1 mM EGTA, 2 mM AEBSF, and EDTA-free Protease Inhibitor Cocktail [Roche]) and lysed by passing through a 26 G × ½-in. needle 15 times. Parasite nuclei were centrifuged at 14,000 × g for 15 min at 4 °C, followed by collection of the supernatant containing the cytoplasmic extract.

### Multidimensional protein identification technology (MudPIT)

Proteins were precipitated with 20 % trichloroacetic acid (TCA). The resulting pellet was washed once with 10 % TCA and twice with cold acetone. The TCA-precipitated protein pellet (about 50 μg) was solubilized in Tris-HCl pH 8.5 and 8 M urea. TCEP (Tris(2-carboxyethyl)phosphine hydrochloride, Pierce) and CAM (chloroacetamide, Sigma) were added to a final concentration of 5 mM and 10 mM, respectively. The protein suspension was digested overnight at 37 °C using Endoproteinase Lys-C at 1:50 w/w (Roche). The sample was brought to a final concentration of 2 M urea and 2 mM CaCl_2_ before performing a second overnight digestion at 37 °C using trypsin (Promega) at 1:100 w/w. Formic acid (5 % final) was added to stop the reactions. The sample was loaded on a split-triple-phase fused-silica micro-capillary column [75] and placed in-line with a linear ion trap mass spectrometer (LTQ) (Thermo Scientific), coupled with a Quaternary Agilent 1260 Series HPLC system. Polysome replicate 1 samples and the ring stage control sample were analyzed on a Velos Pro ion-trap instrument using the LTQ, while all other samples were analyzed using LTQ only. All samples were run in low resolution mode. A fully automated 10-step chromatography run (for a total of 20 h) was carried out, as described in [76]. Each full MS scan (400–1600 m/z) was followed by five data-dependent MS/MS scans. The number of the micro scans was set to 1 both for MS and MS/MS. The dynamic exclusion settings used were as follows: repeat count 2; repeat duration 30 s; exclusion list size 500 and exclusion duration 120 s, while the minimum signal threshold was set to 100. The MS/MS data set was searched using SEQUEST [77] against a database of 72,358 sequences, consisting of 5487 *P. falciparum* non-redundant proteins (downloaded from PlasmoDB on 12 July 2012), 30,536 *H. sapiens* non-redundant proteins (downloaded from NCBI on 27 August 2012), 177 usual contaminants (such as human keratins, IgGs, and proteolytic enzymes), and, to estimate false discovery rates (FDRs), 36,179 randomized amino acid sequences derived from each non-redundant protein entry. To account for alkylation by CAM, 57 Da were added statically to the cysteine residues. To account for the oxidation of methionine residues to methionine sulfoxide (which can occur as an artifact during sample processing), 16 Da were added as a differential modification to the methionine residue. Peptide/spectrum matches were sorted and selected using DTASelect/CONTRAST [78]. Proteins had to be detected by one peptide with two independent spectra, leading to average FDRs at the protein and spectral levels of 0.45 % (range, 0–1.13 %) and 0.12 % (range, 0–0.33 %), respectively, for the interactome capture experiments and 1.26 % (range, 0.15–2.56 %) and 0.09 % (range, 0.01–0.17 %), respectively, for the polysome isolation experiments. To estimate relative protein levels and to account for peptides shared between proteins, normalized spectral abundance factors (dNSAFs) were calculated for each detected protein, as described in [79].

### MudPIT data analysis

A total of four independent mRNA-interactome capture experiments were performed: two biological replicates each for trophozoite-stage and schizont-stage parasites. Enrichment of RBPs in each individual experiment was defined as detection of two or more spectra of that protein in the capture sample and a more than twofold higher normalized abundance (dNSAF) as compared to the control RNase sample. Data from the four independent experiments were then combined, and proteins that were enriched in at least two independent experiments were considered candidate mRBPs. Proteins that were detected at a higher abundance in the control samples than in the corresponding capture sample were considered depleted. Ribosomal proteins were considered contaminants and were removed from the list of detected proteins. Lists of all proteins that were detected in our samples and individual peptide/spectral counts are provided in Additional file 5. The QSpec statistical package (v. 1.2.2) was also used to define a statistically significant list of proteins enriched in the capture experiments (combining replicates from trophozoites and schizonts) compared to controls. Distributed spectral counts and lengths of the detected proteins were inputted to the online interface (http://www.nesvilab.org/qspec.php/). QSpec uses a Bayesian hierarchical model to derive statistical information for estimates of mean and variance across all proteins when limited numbers of replicates are available [80]. Proteins with a log_2_(FoldChange) > 0 and FDRup values <0.05 were considered significantly enriched.

The likelihood that highly abundant proteins were detected as a result of contamination was assessed by comparing the sum of spectral counts from all capture samples to the sum of all spectra from an existing mass spectrometry data set [46] using the chi-square test with Benjamini-Hochberg correction for multiple testing in R v2.7.0.

Polysomes were isolated in duplicate from three parasite stages (ring, trophozoite, schizont). Polysome-associated proteins were defined as proteins that were detected in both series of replicates and that were more than twofold enriched compared to cytoplasmic fractions in at least half of the samples in which the protein was detected. Lists of all proteins that were detected in our samples and individual peptide/spectral counts are provided in Additional file 7.

### GO and Pfam domain enrichment analysis

The enrichment of Gene Ontology (GO) terms was analyzed using the software package topGO (written in R and maintained by the BioConductor project) [81]. For each GO domain (i.e., cellular component, biological process, or molecular function), we compared the proteins identified by MudPIT to the full proteome of *P. falciparum* using the “classic” algorithm in combination with a Fisher’s exact test. GO terms with a *p* value < 0.01 were reported. Enrichment of Pfam domains was tested using the hypergeometric test with Benjamini-Hochberg correction for multiple testing in R v2.7.0, as described in [43].

## Additional files

Additional file 1: List of RNA-binding domains used for HMM searches. (XLSX 67 kb) Additional file 2: List of predicted RNA-binding proteins in *P. falciparum*. (XLSX 112 kb) Additional file 3: Document containing supplementary **Figures S1–S10** and their legends. (PDF 1225 kb) Additional file 4: Comparative analysis of the abundance of RNA-binding domains in various organisms. (XLSX 191 kb) Additional file 5: Experimentally captured RNA-binding proteins at the trophozoite and schizont stages, including mass spectrometry data. (XLSX 1361 kb) Additional file 6: Enriched GO terms and enrichment of mRNA-binding domains among experimentally captured RNA-binding proteins. (XLSX 45 kb) Additional file 7: Proteins associated with polysomes during the IDC, including mass spectrometry data. (XLSX 1246 kb)
