# Supplementary material for: The mRNA-bound proteome of the human malaria parasite Plasmodium falciparum
Source: Genome Biol. 2016 Jul 5;17:147. doi: 10.1186/s13059-016-1014-0 (PMC4933991; doi:10.1186/s13059-016-1014-0)
Supplement: Additional file 3: — Document containing supplementary Figures S1–S10 and their legends. (PDF 1225 kb) [file 13059_2016_1014_MOESM3_ESM.pdf]

Supplementary figures and notes to:

The mRNA-bound proteome of the human malaria parasite *Plasmodium falciparum*

Evelien M. Bunnik, Gayani Batugedara, Anita Saraf, Jacques Prudhomme, Laurence Florens, and Karine G. Le Roch

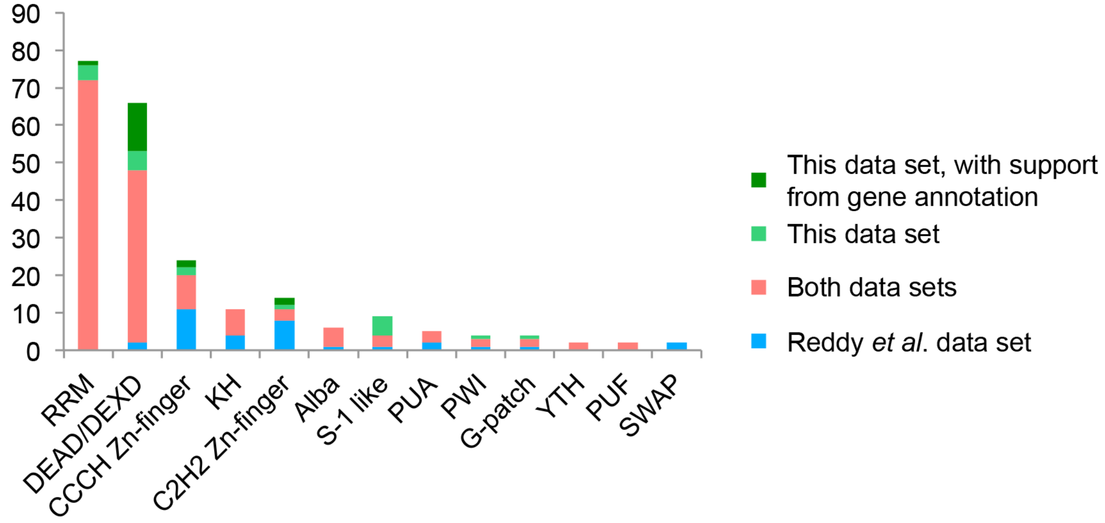

Figure S1: Comparison between HMM search results from this study and Reddy *et al.* [30]. The overlap in proteins between both studies is shown in red, while proteins reported by Reddy *et al.* but not confirmed in this study are indicated in blue. Proteins that were only identified in this study are shown in green, and are further divided based on whether the PlasmoDB gene annotation supports their classification as RNA-binding protein (for RRM), helicase (for DEAD/DEXD), or zinc finger (for C2H2 and CCCH).

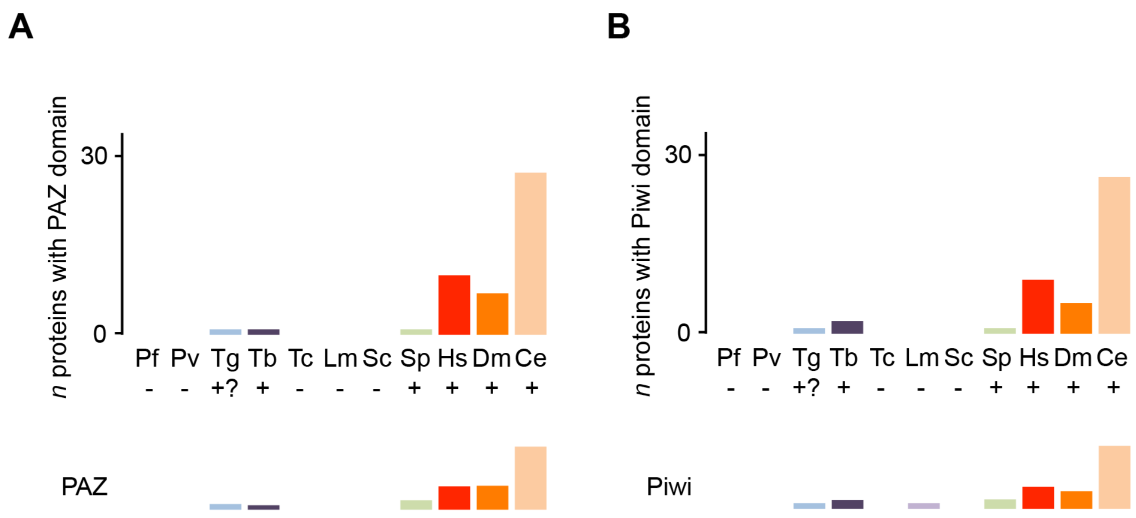

Figure S2: Abundance of mRNA-binding domains found in proteins involved in RNA interference.

(A) Absolute number of proteins with a Piwi Argonaute Zwille (PAZ) domain (top) and the relative frequency of proteins with a PAZ domain (bottom). The mRBD frequency in an organism was first normalized by proteome size and then scaled to the mRBD frequency in the organism with the highest relative abundance of that mRBD. (B) Same as (A) for proteins with a Piwi domain. Organisms are color-coded as in Figure 2.

Pf: *Plasmodium falciparum*, Pv: *Plasmodium vivax*, Tg: *Toxoplasma gondii*, Tb: *Trypanosoma brucei*, Tc: *Trypanosoma cruzii*, Lm: *Leishmania major*, Sc: *Saccharomyces cerevisiae*, Sp: *Saccharomyces pombe*, Hs: *Homo sapiens*, Dm: *Drosophila melanogaster*, Ce: *Caenorhabditis elegans*.

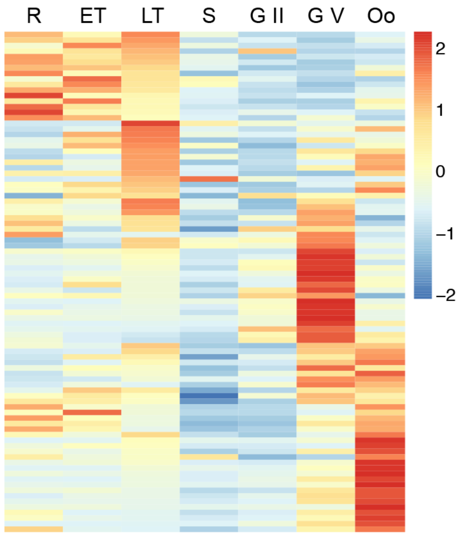

Figure S3: Expression patterns of candidate RNA-binding proteins with RBDs that are exclusively enriched in *Plasmodium* species. RNA-Seq expression data for seven stages of the parasite life cycle [60] is shown for a total of 90 proteins with RBDs that fall into cluster 1 (see Figure 2A). R: ring, ET: early trophozoite, LT: late trophozoite, S: schizont, G II: stage II (early) gametocytes, G V: stage V (late) gametocytes, Oo: ookinete.

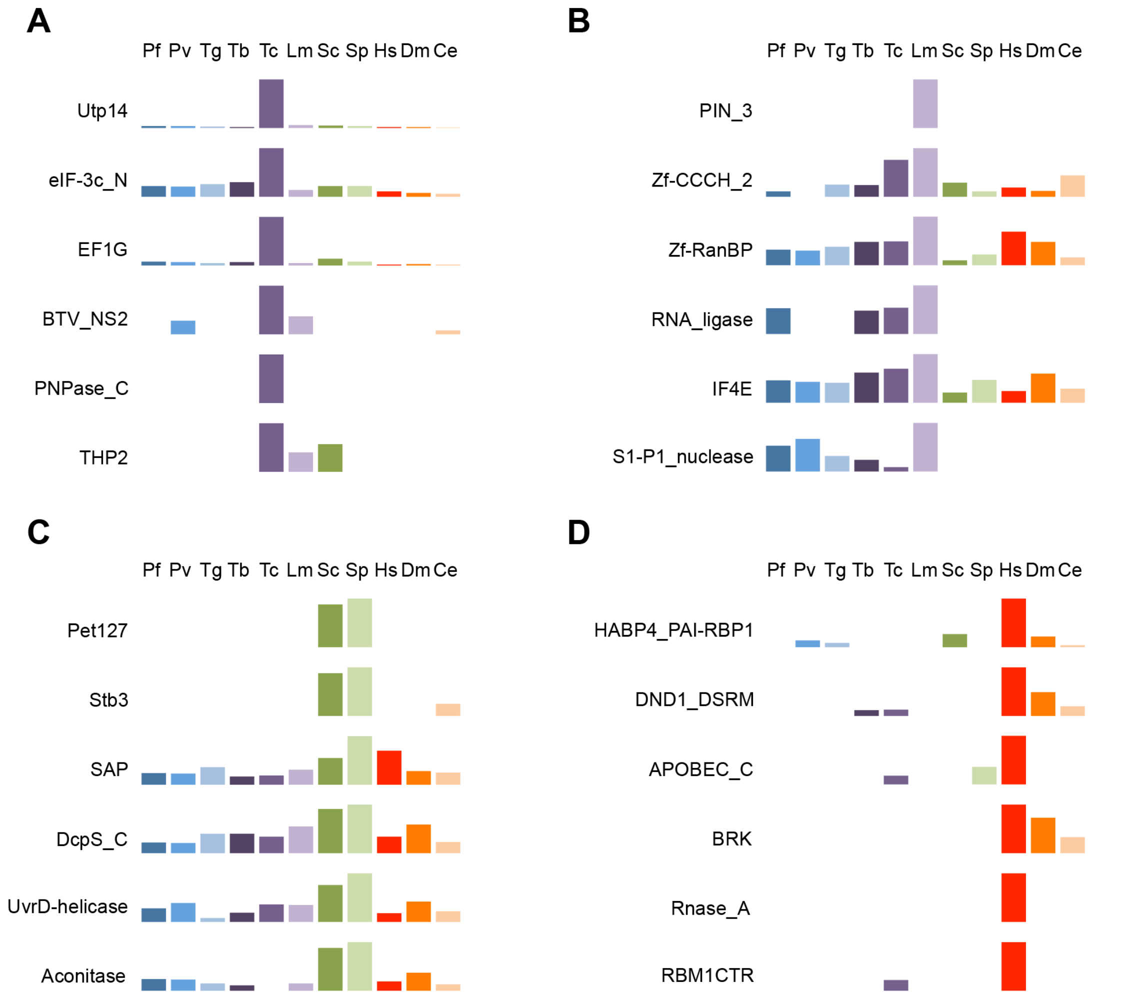

Figure S4: Relative abundance of mRNA-binding domains with species-specific frequency patterns.

(A) mRNA-binding domains that are relatively abundant in *Trypanosoma cruzii* (cluster 4). (B) mRNA-binding domains that are relatively abundant in *Leishmania major* (cluster 5). (C) mRNA-binding domains that are relatively abundant in *Saccharomyces* spp. (cluster 6 or 7). (D) mRNA-binding domains that are relatively abundant in *Homo sapiens* (cluster 8).

The mRBD frequency in an organism was first normalized by proteome size and then scaled to the mRBD frequency in the organism with the highest relative abundance of that mRBD. In all panels, organisms are color-coded as in Figure 2. Pf: *Plasmodium falciparum*, Pv: *Plasmodium vivax*, Tg: *Toxoplasma gondii*, Tb: *Trypanosoma brucei*, Tc: *Trypanosoma cruzii*, Lm: *Leishmania major*, Sc: *Saccharomyces cerevisiae*, Sp: *Saccharomyces pombe*, Hs: *Homo sapiens*, Dm: *Drosophila melanogaster*, Ce: *Caenorhabditis elegans*.

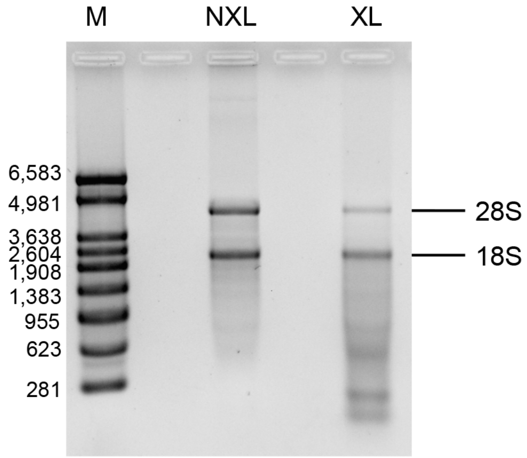

Figure S5: RNA integrity after crosslinking of parasites by UV at 254 nm. Total RNA was extracted from non-crosslinked (NXL) and UV-crosslinked (XL) parasites and was visualized on a 1% agarose gel stained with ethidium bromide. M: marker with the size of RNA bands indicated on the left.

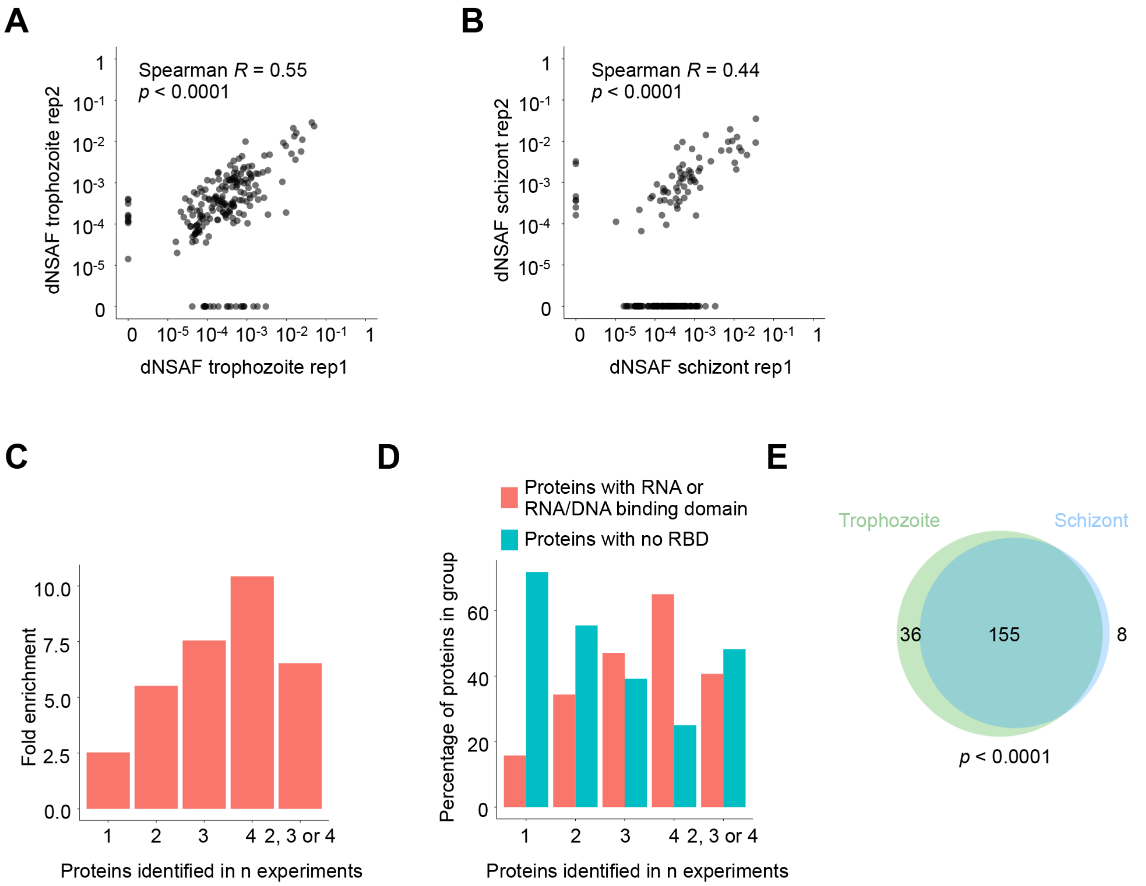

Figure S6: Additional validation of mRNA-interactome capture results.

(A) Correlation in abundance of captured proteins in replicate experiments at the trophozoite stage. (B) Correlation in abundance of captured proteins in replicate experiments at the schizont stage. In panels A and B, proteins that were not detected were given a normalized abundance value of 10^{-6} for plotting purposes. The original value was used to calculate Spearman's correlation coefficient. (C) Fold enrichment of proteins with an RNA or DNA/RNA-binding domain among groups of proteins identified in replicate experiments, as compared to proteins that were depleted in these capture experiments. (D) Percentage of proteins with or without an RNA-binding domain among proteins identified in replicate capture experiments. In both panels, the bars labeled with "2, 3 or 4" are all proteins that were captured in 2 or more experiments and that are considered confident hits. (E) Overlap in the mRNA-bound proteome at the trophozoite and schizont stages for the 199 candidate mRNA-binding proteins that were detected in at least two out of four capture samples. The overlap between groups was assessed using the hypergeometric test.

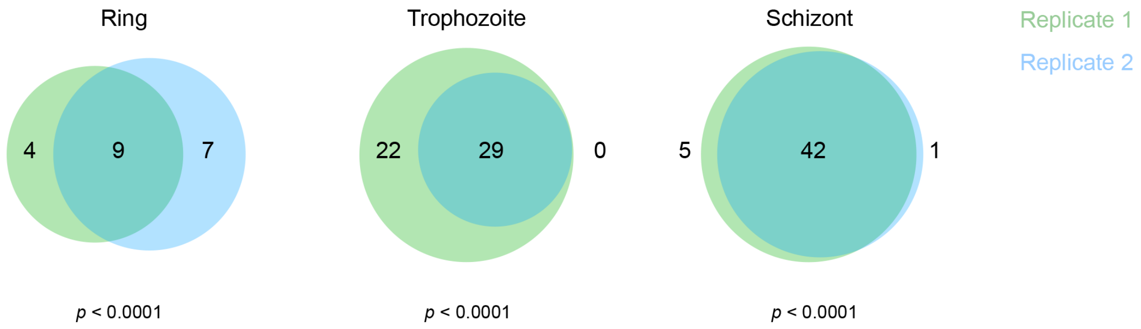

Figure S7: Overlap in non-ribosomal proteins associated with polysomes at the ring (left), trophozoite (middle), and schizont (right) stages. The overlaps between groups were assessed using the hypergeometric test.

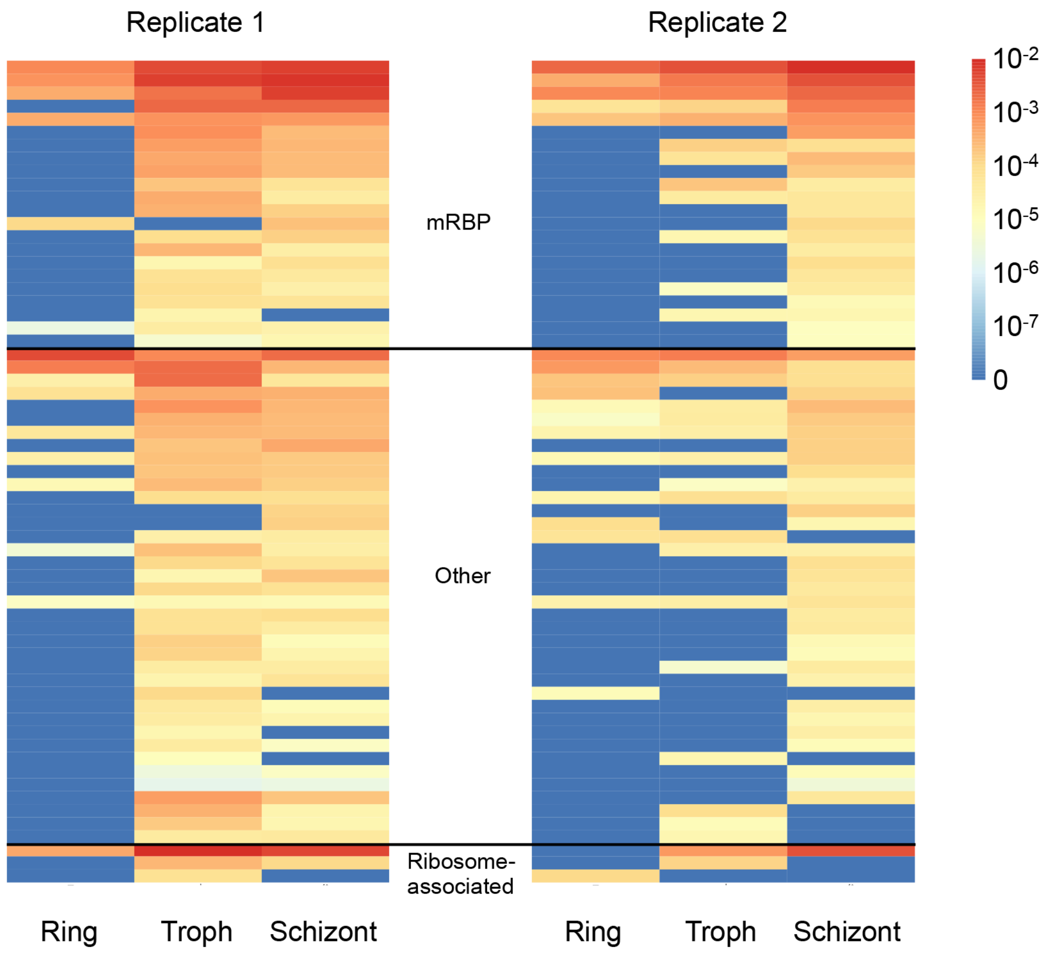

Figure S8: **Abundance of non-ribosomal proteins detected in polysome fractions. Proteins are ranked in the same order as in Figure 4D.**

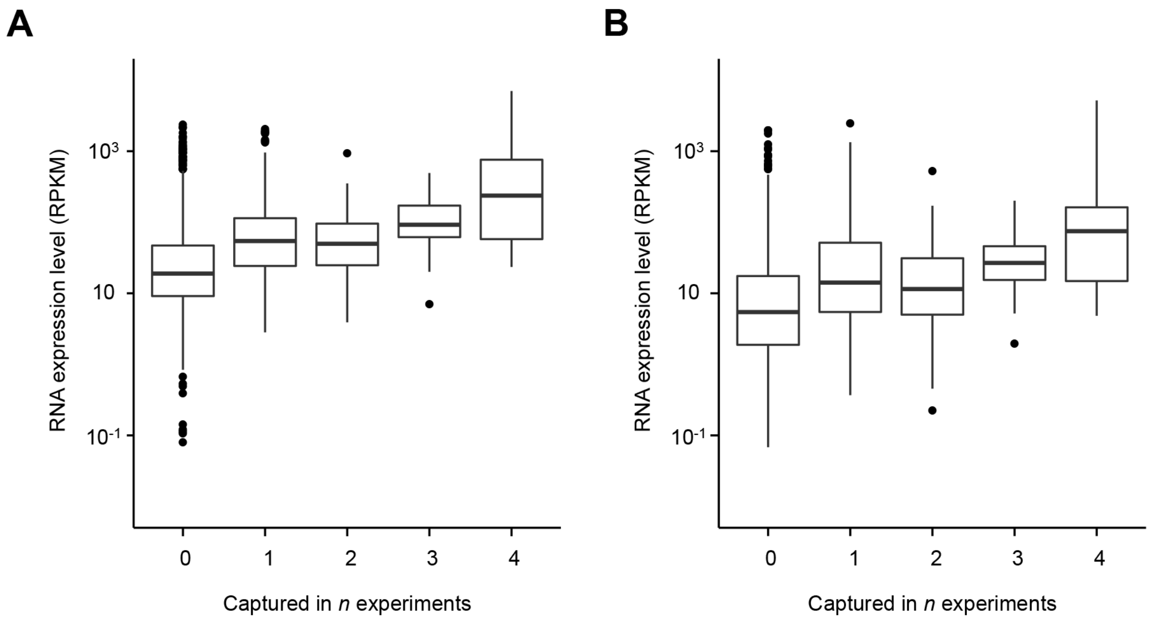

Figure S9: **Relation between RNA-Seq expression levels and detection of candidate RBPs in our capture experiments.** The distribution of RNA-Seq expression levels (RPKM) at the late trophozoite stage (A) or schizont stage (B) is shown for proteins that were not captured (0) or were captured in one, two, three or four independent experiments. Expression data was obtained from a published RNA-Seq data set [60] available through PlasmoDB.

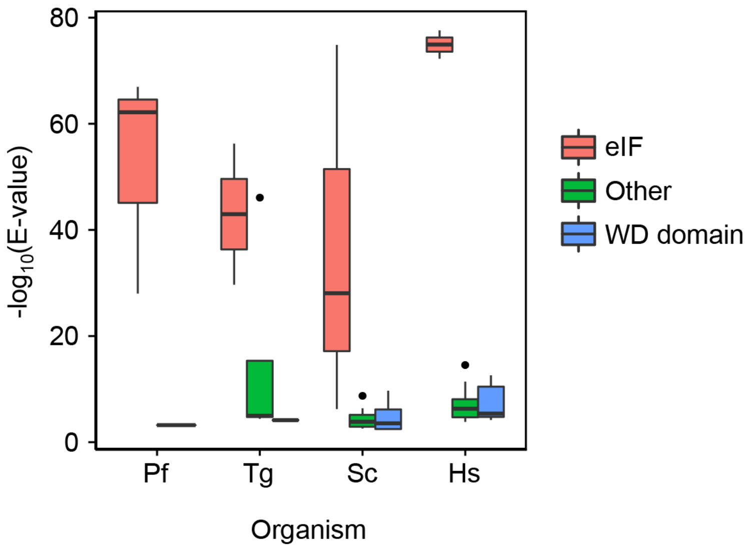

Figure S10: **Distribution of E-values for Pfam domain eIF2A (PF08662) among proteins in four organisms.** The HMM search yielded low E-values (E < 1E-15) for true hits (i.e. eukaryotic translation initiation factors, eIF), but also gave false positive results with E-values between 0.01 and 1E-15, in particular for proteins with a WD domain. To filter out these false positive hits, a more stringent E-value cutoff (1E-15) was used for this particular Pfam domain. Pf: *P. falciparum*, Tg: *T. gondii*, Sc: *S. cerevisiae*, Hs: *H. sapiens*.
